# Effect of nutritionally modified infant formula on academic performance: linkage of seven dormant randomised controlled trials to national education data

**DOI:** 10.1136/bmj-2021-065805

**Published:** 2021-11-11

**Authors:** Maximiliane L Verfürden, Ruth Gilbert, Alan Lucas, John Jerrim, Mary Fewtrell

**Affiliations:** 1 UCL Great Ormond Street Institute of Child Health, London, UK; 2 UCL Institute of Education, London, UK

## Abstract

**Objective:**

To compare differences in academic performance between adolescents who were randomised in infancy to modified or standard infant formula.

**Design:**

Linkage of seven dormant randomised controlled trials to national education data.

**Setting:**

Five hospitals in England, 11 August 1993 to 29 October 2001, and schools in England, September 2002 to August 2016.

**Participants:**

1763 adolescents (425 born preterm, 299 born at term and small for gestational age, 1039 born at term) who took part in one of seven randomised controlled trials of infant formula in infancy.

**Interventions:**

Nutrient enriched versus standard term formula (two trials), long chain polyunsaturated fatty acid (LCPUFA) supplemented versus unsupplemented formula (two trials), high versus low iron follow-on formula (one trial), high versus low sn-2 palmitate formula (one trial), and nucleotide supplemented versus unsupplemented formula (one trial).

**Main outcome measures:**

The primary outcome, determined by linkage of trial data to school data, was the mean difference in standard deviation scores for mandated examinations in mathematics at age 16 years. Secondary outcomes included differences in standard deviation scores in English (16 and 11 years) and mathematics (11 years). Analysis was by intention to treat with multiple imputation for participants missing the primary outcome.

**Results:**

1607 (91.2%) participants were linked to school records. No benefit was found for performance in mathematics examinations at age 16 years for any modified formula: nutrient enriched in preterm infants after discharge from hospital, standard deviation score 0.02 (95% confidence interval −0.22 to 0.27), and nutrient enriched in small for gestational age term infants −0.11 (−0.33 to 0.12); LCPUFA supplemented in preterm infants −0.19 (−0.46 to 0.08) and in term infants −0.14 (−0.36 to 0.08); iron follow-on formula in term infants −0.12 (−0.31 to 0.07); and sn-2 palmitate supplemented formula in term infants −0.09 (−0.37 to 0.19). Participants from the nucleotide trial were too young to have sat their General Certificate of Secondary Education (GCSE) examinations at the time of linkage to school data. Secondary outcomes did not differ for nutrient enriched, high iron, sn-2 palmitate, or nucleotide supplemented formulas, but at 11 years, preterm and term participants randomised to LCPUFA supplemented formula scored lower in English and mathematics.

**Conclusions:**

Evidence from these randomised controlled trials indicated that the infant formula modifications did not promote long term cognitive benefit compared with standard infant formulas.

## Introduction

Breastfeeding is best for infant nutrition, with multiple health benefits for infants and mothers. But the rates for breastfeeding and for continuing breastfeeding beyond six weeks are low in many high and middle income countries.^1^ Infant formulas (breast milk substitutes) are widely used to supplement or replace breastfeeding and might be the sole source of nutrition in the first six months of life. Optimising the composition of these products to meet nutritional requirements and reduce disadvantages in health outcomes between breastfed and formula fed infants is, therefore, an important public health issue. Compositional standards for infant formulas are specified by the Codex Alimentarius^2^ and, in the European Union, regulated by the European Directive^3^ based on the composition of breast milk, estimated requirements, and evidence from randomised controlled trials on short and long term benefits.^4^ One goal of modifying infant formula is to make long term cognitive outcomes more like those seen in breastfed infants.

In this study, we discuss three types of modified infant formulas, which are widely available and have been proposed to promote cognitive development: formula enriched with nutrients, formula supplemented with long chain polyunsaturated fatty acids (LCPUFAs), and follow-on formula fortified with iron. Nutrient enriched formulas were designed to meet the additional nutritional requirements of preterm babies after discharge from hospital and to support catch-up growth in infants born small for gestational age at term, and thus help cognitive development.^5^ LCPUFAs have been added to preterm and term infant formulas with the aim of improving visual and cognitive outcomes.^6^ ^7^ The addition of one type of LCPUFA, docosahexaenoic acid, has recently been mandated in the European Union.^3^ Iron is added to follow-on formulas to improve iron status during the complementary feeding period when infants have high requirements for normal growth and development, with the aim of avoiding the adverse effects of iron deficiency on cognitive development.^8^

Results from previous randomised controlled trials of each of the three types of modifications were inconclusive for cognitive ability.^5^ ^6^ ^7^ ^9^ Evidence was based mainly on developmental assessments before the age of 2 years, which are inherently noisy and poorly predictive of later outcomes.^10^ Because cognitive function changes and matures over time in children, the hypothesis that cognitive differences might become more apparent with time was proposed.^4^ A recent randomised controlled trial reported adverse effects of iron fortified follow-on formula on cognitive outcomes at ages 10 and 16 years, despite no evidence of an effect in infancy.^11^ ^12^ But this result is difficult to interpret because of high rates of attrition, which are commonly seen in long term follow-up studies of infant nutrition (supplement 1). 

In this study, we dealt with the problem of attrition by linking, with legal approval and without the need for patient consent, a series of randomised controlled trials of infant formulas,^9^ ^13^ ^14^ ^15^ ^16^ covering the three types of formula modifications, to national pupil records: nutrient enriched formula (two trials), LCPUFA supplemented formula (two trials), and iron fortified follow-on formula (one trial). Two more trials of modified infant formula were approved to be linked, although these modifications were not originally hypothesised to improve cognitive ability^17^: one trial involved a formula supplemented with nucleotides^18^ and one a formula supplemented with sn-2 palmitate.^19^

The seven trials were conducted by our group between 1993 and 2001 and are referred to as dormant because the primary outcome for these studies has already been collected and further active follow-up was discontinued; however, participant identifiers were retained enabling reactivation of the trials through linkage. The null hypothesis for all trials was that in the population of children studied, no difference existed between the modified and standard formula groups in academic performance. The objective of this study was to establish whether the trial results support the null hypothesis or provide evidence of a difference between the modified and standard formula groups.

## Methods

### Study design and participants

All seven trials were dormant randomised controlled double blind superiority trials conducted by the authors (AL and MF). Participants were recruited in England between 11 August 1993 and 29 October 2001. The acronyms of the seven trials describe the formula modification and the infant population (box 1). Table 1 summarises the study design and eligibility criteria, which are reported in detail elsewhere.^9^ ^13^ ^14^ ^15^ ^16^ ^18^ ^19^ Supplement 2 shows the primary outcomes for the original trials and the corresponding power calculations. In this study, follow-up of participants was achieved by linking trial data to routinely collected data from the National Pupil Database, collated for all children attending state school in England and all children sitting national curriculum tests, such as the General Certificate of Secondary Education (GCSE). Analysis was by intention to treat for all randomised participants who survived.

Box 1 **Definition of trial acronyms**

NEP=nutrient enriched post discharge formula for babies born preterm
NETSGA=nutrient enriched term formula for babies born at term and small for gestational age
LCPUFAP=preterm formula supplemented with long chain polyunsaturated fatty acids (0.17% docosahexaenoic acid + 0.31% arachidonic acid) for babies born preterm
LCPUFAT=term formula supplemented with long chain polyunsaturated fatty acids (0.32% docosahexaenoic acid + 0.30% arachidonic acid) for babies born at term
IRONT=term follow-on formula with high iron content (12 mg/L of iron) for babies born at term
PALMT=term formula with 50% sn-2 palmitate for babies born at term
NUCLEOT=term formula with 31 mg/L of nucleotides added for babies born at term

**Table 1:** Characteristics of randomised controlled trials included in this study

Trial (first publication)							
	NEP^15^	NETSGA^16^	LCPUFAP^17^	LCPUFAT^18^	IRONT^11^	PALMT^46^	NUCLEOT^47^
**Study design**							
Population	Preterm infants: bw <1750 g, <37 weeks ga	SGA term infants: bw <10th ‎centile‎‎, ≥37 weeks ga	Preterm infants: bw ≤1850 g, ≤37 weeks ga	Term infants:‎ ≥37 weeks ga	Term infants: bw >2500 g, ≥37 weeks ga, age: 9 months	Term infants: ≥37 weeks ga, bw >5th centile	Term infants: ≥37 weeks ga
Formula modification	Nutrient enriched (mainly protein and calorie) term formula (after discharge)	Nutrient enriched (mainly protein and calorie) term formula	Preterm formula supplemented with 0.17% DHA + 0.31% AA	Term formula supplemented with 0.32% DHA + 0.30% AA	Term formula with 12 mg/L iron	Term formula supplemented with 50% sn-2 palmitate	Term formula supplemented with 31 mg/L nucleotides
Standard formula	Term formula (after discharge)	Standard term formula	Standard preterm formula (without DHA + AA)	Standard term formula (without DHA + AA)	Standard term formula with 0.9 mg/L iron	Standard term formula with 12% sn-2 palmitate	Standard term formula with <5 mg/L nucleotides
Randomisation period	7 October 1993-19 November to 1996	21 September 1993-11 January 1996	11 August 1993-11 May 1996	2 November 1993-30 June 1995	26 October 1993-21 May 1995	15 May 1995-26 November 1996	11 February 2000-29 October 2001
Place of recruitment	NT, L, C, I	NT, L, C	NT, L	NT, L	NT, L, NW	C	NT, L
Start and duration of intervention	Week before planned hospital discharge*, 9 months	Birth, 9 months	Birth, 3 weeks	Birth, 6 months	9, 18 months	Birth, 12 weeks	Birth, 20 weeks
No of contacts per participant (mean (SD))	5.0 (1.5)	4.8 (1.9)	1.7 (0.8)	4.1 (1.8)	5.5 (1.0)	3.1 (0.8)	2.7 (0.9)
**Risk of bias**							
Random sequence generation	Yes	Yes	Yes	Yes	Yes	Yes	Yes
Allocation concealment	Yes	Yes	Yes	Yes	Yes	Yes	Yes
Blinding	Yes	Yes	Yes	Yes	Yes	Yes	Yes
Formula supplier	Farley (now Heinz)	Farley (now Heinz)	Milupa (now Danone)	Nestlé	Wyeth (now part of Nestlé)	Nutricia (Danone)	Heinz
Role of formula supplier in initial trial	Supply of formula, financial assistance	Supply of formula, financial assistance	Supply of formula, financial assistance	Supply of formula, financial assistance	Supply of formula, financial assistance	Supply of formula, financial assistance	Supply of formula, financial assistance

SD=standard deviation; bw=birth weight; ga=gestational age; DHA=docosahexaenoic acid; AA=arachidonic acid; NT=Nottingham; L=Leicester; C=Cambridge; I=Ipswich; NW=Norwich.

Trials: NEP=nutrient enriched post-discharge formula for babies born preterm; NETSGA=nutrient enriched term formula for babies born at term and small for gestational age; LCPUFAP=preterm formula supplemented with long chain polyunsaturated fatty acids (0.17% DHA + 0.31% AA) for babies born preterm; LCPUFAT=term formula supplemented with long chain polyunsaturated fatty acids (0.32% DHA + 0.30% AA) for babies born at term; IRONT=term follow-on formula with high iron content (12 mg/L iron) for babies born at term; PALMT=term formula with sn-2 palmitate for babies born at term; NUCLEOT=term formula with added nucleotides for babies born at term.

* In the NEP trial, week before planned hospital discharge corresponds to the expected date of delivery if the infant had not been born prematurely (term equivalent age).

### Randomisation and masking

In each trial, participants were randomly assigned to a modified or standard formula. Standard formulas corresponded to regulations and practices for the five different trial populations (preterm after discharge from hospital, term and small for gestational age from birth, preterm from birth, term from birth, and term follow-on) at the time, and therefore differed between trials. In each trial, randomisation was performed with permuted blocks of randomised length and prepared by an independent statistician who had no further involvement in the trial. Depending on the trial, randomisation was stratified by centre or birth characteristics, or both. Allocation was concealed with sealed, opaque, numbered envelopes. Formulas were identical in colour and smell and, depending on the trial, identified by a barcode or a colour code held by the formula manufacturers and not revealed to the investigators until after the principal data analyses were performed. Schools were not informed about participation in the trial or group allocation. For all analyses involving academic performance, group allocation was replaced by a dummy number until the analysis plan had been externally peer reviewed. Participants, their parents, the outcome assessor (school examination boards), and the data analyst were also blind to the allocated groups.

### Procedures

Table 1 summarises the details of the interventions, and participant ages at the start and end of each intervention. Supplement 3 details the nutritional compositions of the infant formulas. De-identified trial data, containing all follow-up assessments and intervention allocations, were retrieved from records held by the original trial investigators (AL and MF). For follow-up of routinely collected administrative education records, we linked trial data to education data in the following steps. Firstly, we transcribed information from paper records containing all participant identifiers (name, postcode history, and date of birth) for each trial, from enrolment and for every follow-up contact, onto a database. Each participant was allocated a unique study identifier. Postcodes were checked against a national look-up table, and all names and addresses were standardised. Secondly, the identifier database was transferred by a secure link to a trusted third party, the Fischer Family Trust,^20^ a non-profit organisation commissioned by schools and the Department for Education in England to analyse data from the National Pupil Database. Thirdly, the full chronology of postcodes, with names and dates of birth, were linked deterministically by the Fischer Family Trust to identifiers held in the National Pupil Database. Identifiers used in the National Pupil Database were recorded at the annual spring school census for all pupils in state school in England or at GCSE examinations for any pupil (state or private school) sitting the examination in England. 

Even in true matches, not all identifiers always agree exactly (eg, because of different name spellings). Therefore, the Fischer Family Trust generated up to four candidate links for each trial participant. Fourthly, the candidate links were returned together with the unique study identifier and the administrative school data, but no personal identifiers, to the University College London (UCL) Data Safe Haven, an ISO27001 certified secure online environment maintained by UCL. All candidate links contained information on the linkage strength of each identifier (eg, exact postcode match *v* match to neighbouring authority). The unique study identifier was used to link the trial data to the pupil candidate matches within the UCL Data Safe Haven. Finally, probabilistic methods were used to select the best matching candidate record for each participant, with the information provided by the Fischer Family Trust on linkage strength, and data from the original clinical databases, such as information on death and sibling participants (twins and triplets) in the randomised controlled trial, which the Fischer Family Trust did not have access to. 

The data analyst was unblinded after the trial protocol was submitted and peer reviewed. The total number of randomised participants in this study is based on the original clinical databases rather than the available identifiers, and therefore two more participants were included in this study and erroneous information on participant death was corrected. This study does not include the cow’s milk control group recruited in the IRONT study. This study also states the total number of linked participants as the number after data cleaning, which was not available when the protocol was published. Further information on the linkage and data cleaning process is published elsewhere, including in a code repository.^17^ ^21^ ^22^

### Outcomes

In England, national examinations, known as GCSEs, are mandatory at age 16 years, and results are centrally collated by the Department for Education. Evidence from cohort studies indicates that GCSE grades are strongly predictive of higher education and employment outcomes.^23^ ^24^ Examinations in the study periods were based on the same curriculum, tested the same skills, and were designed by the same examination boards. The prespecified primary outcome was the standardised difference within trials in mathematics score at age 16 years. We used standardised rather than raw scores to allow comparisons with the external literature and with previous measures of cognitive ability collected in the trials. We used examination results corrected for grade inflation, obtained from the Fischer Family Trust, to calculate the standardised scores. We chose mathematics as the primary outcome over English because examination results for mathematics are considered to be graded less subjectively.^25^ We chose the primary endpoint at age 16 rather than earlier ages because age 16 is a stronger predictor of future opportunities in education and in the labour market.^23^ Primary outcome data were not available for one trial (NUCLEOT) because participants were not old enough to have sat the GCSE examinations at the time that the trial and school data were linked. 

The prespecified secondary outcomes were: mean difference in standard deviation scores for GCSE English at age 16, and for mathematics and English at age 11 (final year of primary school); odds ratio of receiving five or more GCSEs at grade C or above (including mathematics and English) in the intervention group compared with controls (≥5 GCSEs at ≥grade C is used in academic performance tables); and the odds ratio of being allocated special educational needs support at any age. We also report the correlation with previously measured outcomes related to developmental or cognitive ability in the trials, published and unpublished, to assess the consistency of measures throughout childhood (supplement 4).

### Statistical analysis

Assuming 80% power, the sample sizes linked to GCSE results allowed detection of standard deviation differences of 0.32 (IRONT), 0.33 (LCPUFAT), 0.33 (NETSGA), 0.37 (NEP), 0.41 (PALMT), and 0.43 (LCPUFAP) in the primary outcome (supplement 2). Because of the scalability of the intervention,^26^ and the age when academic performance was assessed,^27^ these effect sizes were regarded as clinically significant. For mean differences, we used linear regression, and for odds ratios we used logistic regression. To increase the statistical efficiency of the analysis, we adjusted for covariates which were measured before randomisation and selected a priori from evidence of associations with academic performance: maternal smoking, level of maternal education, infant sex, birth weight, gestational age,^28^ ^29^ and study centre.^30^ To account for slightly heteroscedastic residuals (where the variance of residuals was not constant around the regression line), we applied robust standard errors with the sandwich variance estimator at the individual level in all analyses (supplement 5).^22^ Missing covariates, and primary and secondary outcomes, were imputed with chained multiple imputation (n=15) unless the trial record indicated that the child died.^17^ The primary analysis was an adjusted intention-to-treat analysis of the primary outcome, the mean difference in standard deviation score (based on the distribution within each trial) in mathematics at age 16 years.

### Sensitivity analyses

To understand whether the results from the primary analyses were sensitive to small baseline imbalances, missing data, or selection of standardisation reference distribution, sensitivity analyses were performed: unadjusted multiple imputation analysis, adjusted complete case analysis (with the same a priori specified covariates but excluding participants with missing data), unadjusted complete case analysis, and external standardisation to the national grade average from the years 2008-09 to 2011-12. Similar mean differences in all of these analyses would suggest that the results are likely to be robust to observed baseline imbalances, missing data strategy, and choice of standardisation reference distribution. Data cleaning and statistical analyses were done in Stata version 16. All code for data cleaning and analyses, including sensitivity analyses, is publicly available.^22^

### Patient and public involvement

Six members of the public (born preterm and former participants in an infant formula trial) were involved in the conceptualisation stage of this research.

## Results

**Fig 1:**
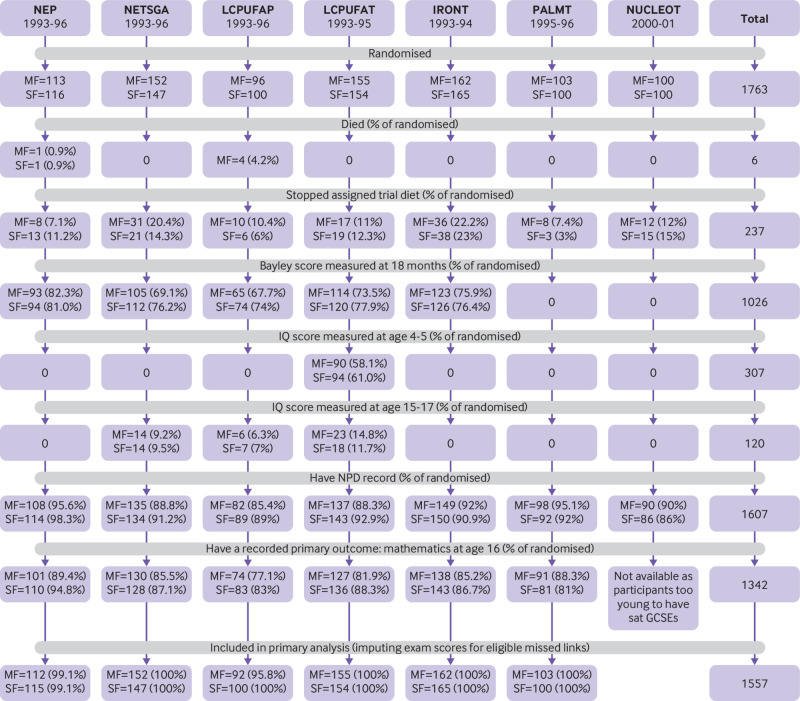
Consort diagram showing the flow of participants by trial and formula group, including previously measured cognitive outcomes. MF=modified formula; SF=standard formula; NPD= National Pupil Database; GCSE=General Certificate of Secondary Education. Trials: NEP=nutrient enriched post-discharge formula for babies born preterm; NETSGA=nutrient enriched term formula for babies born at term and small for gestational age; LCPUFAP=preterm formula supplemented with long chain polyunsaturated fatty acids (0.17% docosahexaenoic acid (DHA) + 0.31% arachidonic acid (AA)) for babies born preterm; LCPUFAT=term formula supplemented with long chain polyunsaturated fatty acids (0.32% DHA + 0.30% AA) for babies born at term; IRONT=term follow-on formula with high iron content (12 mg/L iron) for babies born at term; PALMT=term formula with sn-2 palmitate for babies born at term; NUCLEOT=term formula with added nucleotides for babies born at term

Figure 1 shows that of 1763 randomised participants, 91.2% (n=1607) were linked to a pupil record in the National Pupil Database. In the six trials (n=1563) where all participants were old enough to have sat their GCSE examinations, 85.9% (n=1342) were linked to a GCSE result in mathematics. Baseline characteristics for the linked participants (table 2) were mostly balanced across the trial groups. Exceptions were that in the NEP trial, infants in the intervention group were less likely to have a mother with a degree (6% *v* 12%, P=0.131); in the LCPUFAP trial, infants in the intervention group were more likely to have mothers who smoked during pregnancy (44% *v* 39%, P=0.211); and in the LCUPFAT trial, infants in the intervention group were more likely to have a mother with a degree (8% *v* 4%, P=0.169). Supplement 6 gives the baseline characteristics at randomisation.

**Table 2:** Participant characteristics at randomisation in the randomised controlled trials linked to the National Pupil Database

	NEP			NETSGA			LCPUFAP			LCPUFAT			IRONT			PALMT			NUCLEOT	
	MF	SF		MF	SF		MF	SF		MF	SF		MF	SF		MF	SF		MF	SF
Linked/randomised*	108/113	114/116		135/152	134/147		82/96	89/100		137/155	143/154		149/162	150/165		98/103	92/100		90/100	86/100
Birth weight (g)	1392 (775-2160)	1363 (630-2020)		2538 (1400-3160)	2597 (1770-3160)		1351 (640-1850)	1344 (740-1780)		3654 (2960-4900)	3532 (2680-4930)		3483 (2495-5046)	3468 (2466-4706)		3588 (2460-4730)	3472 (2520-5400)		3453 (2210-4720)	3482 (2170-5360)
Gestational age (weeks)	30.8 (26-36)	30.8 (25-36)		39.1 (37-42)	39.4 (37-42)		30.5 (25-36)	30.1 (25-36)		40.1 (37-42)	40.0 (37-42)		39.7 (36-43)	39.9 (35-43)		40.1 (37-42)	39.9 (37-42)		39.5 (37-42)	39.2 (37-42)
Mother’s age	28.0 (16-41)	28.5 (17-44)		26.9 (15-42)	26.6 (16-42)		26.2 (16-39)	26.6 (17-39)		27.8 (17-44)	27.0 (18-41)		27.8 (17-40)	27.6 (15-39)		27 (15-40)	28 (17-42)		27 (16-38)	27 (16-40)
Sex:																				
Male	51 (47)	56 (49)		68 (50)	63 (47)		38 (46)	47 (53)		73 (53)	77 (54)		76 (51)	76 (51)		62 (63)	46 (50)		55 (61)	47 (55)
Female	57 (53)	58 (51)		67 (50)	71 (53)		44 (53)	42 (47)		64 (47)	66 (46)		73 (49)	74 (49)		36 (37)	46 (50)		36 (39)	39 (45)
Mother smoked during pregnancy:																				
No	64 (62)	74 (68)		69 (55)	62 (51)		46 (56)	54 (61)		102 (76)	104 (75)		108 (73)	104 (71)		62 (63)	69 (75)		65 (73)	51 (60)
Yes	38 (38)	35 (32)		57 (45)	59 (49)		36 (44)	35 (39)		32 (24)	35 (25)		40 (27)	43 (29)		36 (37)	23 (25)		24 (27)	34 (40)
Missing (No)	4	5		9	13		0	0		3	4		1	3		0	0		1	1
Mother has degree:																				
No	102 (94)	96 (88)		130 (96)	127 (95)		49 (92)	44 (92)		124 (92)	135 (96)		130 (88)	136 (92)		†	†		83 (92)	77 (92)
Yes	6 (6)	13 (12)		5 (4)	6 (5)		4 (8)	4 (8)		11 (8)	6 (4)		18 (12)	12 (8)		†	†		7 (8)	7 (8)
Missing (No)	0	5		0	1		29	41		2	2		1	2		71	73		0	2

Data are mean (minimum-maximum) or number (%), unless indicated otherwise.

MF=modified formula, SF=standard formula; DHA=docosahexaenoic acid; AA=arachidonic acid.

Trials: NEP=nutrient enriched post-discharge formula for babies born preterm; NETSGA=nutrient enriched term formula for babies born at term and small for gestational age; LCPUFAP=preterm formula supplemented with long chain polyunsaturated fatty acids (0.17% DHA + 0.31% AA) for babies born preterm; LCPUFAT=term formula supplemented with long chain polyunsaturated fatty acids (0.32% DHA + 0.30% AA) for babies born at term; IRONT=term follow-on formula with high iron content (12 mg/L iron) for babies born at term; PALMT=term formula with sn-2 palmitate for babies born at term; NUCLEOT=term formula with added nucleotides for babies born at term.

* Number/total randomised study population (irrespective of survival).

† Suppressed because of low numbers.

### Primary outcome

**Fig 2:**
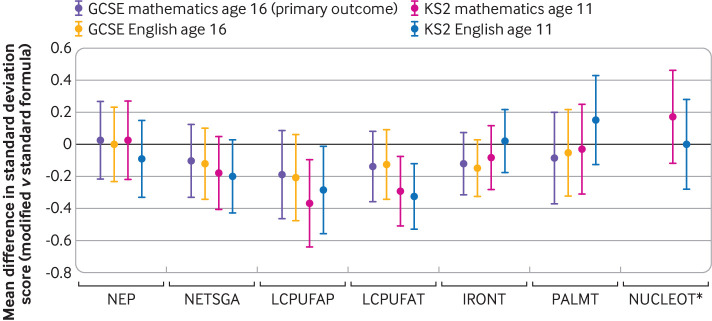
Primary and secondary analysis results: mean differences in internally standardised grade scores in modified versus standard formula groups (negative mean difference=modified formula group performed poorer compared with trial average score). GCSE=General Certificate of Secondary Education; KS=key stage. Trials: NEP=nutrient enriched post-discharge formula for babies born preterm; NETSGA=nutrient enriched term formula for babies born at term and small for gestational age; LCPUFAP=preterm formula supplemented with long chain polyunsaturated fatty acids (0.17% docosahexaenoic acid (DHA) + 0.31% arachidonic acid (AA)) for babies born preterm; LCPUFAT=term formula supplemented with long chain polyunsaturated fatty acids (0.32% DHA + 0.30% AA) for babies born at term; IRONT=term follow-on formula with high iron content (12 mg/L iron) for babies born at term; PALMT=term formula with sn-2 palmitate for babies born at term; NUCLEOT=term formula with added nucleotides for babies born at term. *GCSE data for NUCLEOT are not displayed because most participants were not old enough to have sat their GCSE examinations at the time of linkage

**Fig 3:**
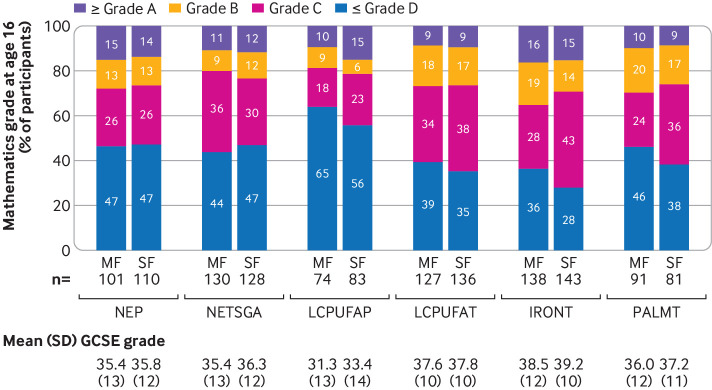
Grade distribution at age 16 years, by trial and trial arm (complete case data). MF=modified formula, SF=standard formula; SD=standard deviation; GCSE=General Certificate of Secondary Education. Trials: NEP=nutrient enriched post-discharge formula for babies born preterm; NETSGA=nutrient enriched term formula for babies born at term and small for gestational age; LCPUFAP=preterm formula supplemented with long chain polyunsaturated fatty acids (0.17% docosahexaenoic acid (DHA) + 0.31% arachidonic acid (AA)) for babies born preterm; LCPUFAT=term formula supplemented with long chain polyunsaturated fatty acids (0.32% DHA + 0.30% AA) for babies born at term; IRONT=term follow-on formula with high iron content (12 mg/L iron) for babies born at term; PALMT=term formula with sn-2 palmitate for babies born at term. GCSE data for the NUCLEOT (term formula with added nucleotides for babies born at term) study are not displayed because most participants were not old enough to have sat their GCSE examinations at the time of linkage

Results for the primary outcome showed that none of the modified formulas improved scores for mathematics at age 16 years (fig 2 and table 3). The adjusted standardised mean difference favoured reduced scores for GCSE mathematics in the modified formula group in all trials except the NEP trial. Lower 95% confidence limits included moderate reductions in scores for mathematics, and upper 95% confidence limits excluded moderate to large benefits for all formula modifications: NEP 0.02 standard deviation (95% confidence interval −0.22 to 0.27); NETSGA −0.11 (−0.33 to 0.12); LCPUFAP −0.19 (−0.46 to 0.08); LCPUFAT −0.14 (−0.36 to 0.08); and IRONT −0.12 (−0.31 to 0.07). As expected for PALMT, we found no benefit or harm of the formula on academic performance on the primary outcome (−0.09 (−0.37 to 0.19)). Participants in the NUCLEOT trial were too young to have sat their GCSE examinations at the time of data linkage. Sensitivity analyses showed that all findings were robust to covariate adjustment and methods of handling missing data (supplement 7). Figure 3 shows that, as expected, trials involving participants born preterm or at term and small for gestational age had lower scores for GCSE mathematics in both trial arms compared with trials involving participants born healthy at term.

**Table 3:** Primary analysis of the primary outcome, with mean and standard deviation of standardised GCSE mathematics grade within trials in the modified and standard formula groups, and their mean differences with 95% confidence intervals

Trial	Population	Modified formula group				Standard formula group				Primary outcome* (MI, adjusted; standardised mean difference (95% CI))
		Formula	Mean SD score	SD		Formula	Mean SD score	SD		
NEP	Preterm	Nutrient enriched term formula (after discharge)	0.01	0.95		Standard term formula after discharge	−0.01	0.92		0.02 (−0.22 to 0.27)
NETSGA	Term SGA	Nutrient enriched term formula	−0.05	0.97		Standard term formula	0.05	0.99		−0.11 (−0.33 to 0.12)
LCPUFAP	Preterm	Preterm formula supplemented with long chain polyunsaturated fatty acids (0.17% DHA + 0.31% AA)	−0.10	0.89		Standard preterm formula (w/o added DHA + AA)	0.09	0.97		−0.19 (−0.46 to 0.08)
LCPUFAT	Term	Term formula supplemented with long chain polyunsaturated fatty acids (0.32% DHA + 0.30% AA)	−0.07	0.97		Standard term formula (w/o added DHA + AA)	0.07	0.97		−0.14 (−0.36 to 0.08)
IRONT	Term	Term formula with high iron content (12 mg/L iron)	−0.06	0.95		Standard term formula (0.9 mg/L iron)	0.06	0.83		−0.12 (−0.31 to 0.07)
PALMT	Term	Term formula with 50% sn-2 palmitate	−0.04	1.03		Standard term formula (12% sn-2 palmitate)	0.05	0.96		−0.09 (−0.37 to 0.19)

SGA=small for gestational age; GCSE=General Certificate of Secondary Education; MI=multiple imputation; SD=standard deviation; DHA=docosahexaenoic acid; AA=arachidonic acid; w/o=without.

Adjusted for infant sex, birth weight, gestational age, recruitment centre, maternal smoking during pregnancy, and maternal education at birth.

Supplement 7 provides details of sensitivity analyses.

Trials: NEP=nutrient enriched post-discharge formula for babies born preterm; NETSGA=nutrient enriched term formula for babies born at term and small for gestational age; LCPUFAP=preterm formula supplemented with long chain polyunsaturated fatty acids (0.17% DHA + 0.31% AA) for babies born preterm; LCPUFAT=term formula supplemented with long chain polyunsaturated fatty acids (0.32% DHA + 0.30% AA) for babies born at term; IRONT=term follow-on formula with high iron content (12 mg/L iron) for babies born at term; PALMT=term formula with sn-2 palmitate for babies born at term; NUCLEOT=term formula with added nucleotides for babies born at term.

* Primary outcome is standardised GCSE mathematics grade within trials.

### Secondary outcomes

Secondary outcomes were consistent with the effect direction of the primary analyses (fig 2 and supplement 7). Infants randomised to the LCPUFA supplemented formula had significantly reduced scores for mathematics at age 11 years (preterm −0.37 standard deviation, 95% confidence interval −0.64 to −0.09, P=0.009 and term −0.29, −0.51 to −0.08, P=0.008), and similar reductions for English at 11 years (preterm −0.29, −0.56 to −0.01, P=0.043 and term −0.33, −0.53 to −0.12, P=0.002). We found no differences in the odds of being eligible for special educational needs support or attaining five or more GCSEs at grade C or above (table 4).

**Table 4:** Secondary outcomes: odds ratios of receiving five or more GCSEs at ≥grade C (including mathematics and English) and of ever being eligible for special educational needs support in modified versus standard formula groups

Trial	Ever eligible for special educational needs support (OR (95% CI))	Achieved ≥5 GCSEs ‎at ≥grade C (OR (95% CI))
NEP	1.29 (0.72 to 2.32)	1.27 (0.70 to 2.29)
NETSGA	1.49 (0.90 to 2.47)	1.00 (0.60 to 1.71)
LCPUFAP	1.34 (0.68 to 2.64)	0.65 (0.32 to 1.31)
LCPUFAT	1.29 (0.78 to 2.14)	0.69 (0.41 to 1.16)
IRONT	1.32 (0.80 to 2.18)	0.89 (0.54 to 1.48)
PALMT	0.81 (0.42 to 1.53)	1.30 (0.67 to 2.52)
NUCLEOT	0.50 (0.25 to 1.01)	*

GCSE=General Certificate of Secondary Education; OR=odds ratio.

Adjusted for sex, birth weight, gestational age, recruitment centre, maternal smoking during pregnancy, and maternal education at birth, covariates and outcomes imputed for missing participants who have not died,

Trials: NEP=nutrient enriched post-discharge formula for babies born preterm; NETSGA=nutrient enriched term formula for babies born at term and small for gestational age; LCPUFAP=preterm formula supplemented with long chain polyunsaturated fatty acids (0.17% DHA + 0.31% AA) for babies born preterm; LCPUFAT=term formula supplemented with long chain polyunsaturated fatty acids (0.32% DHA + 0.30% AA) for babies born at term; IRONT=term follow-on formula with high iron content (12 mg/L iron) for babies born at term; PALMT=term formula with sn-2 palmitate for babies born at term; NUCLEOT=term formula with added nucleotides for babies born at term.

* Data not available because most participants from the NUCLEOT study were not old enough to have sat their GCSE examinations at the time of linkage.

### Consistency with earlier assessments

Results for academic performance were consistent with earlier assessments of cognitive ability in the analysed trials: Bayley scales at age 18 months^9^ ^13^ ^14^ ^15^ ^16^ indicated no evidence of benefit of the modified formulas. Unpublished data from the trials showed significantly reduced Wechsler IQ scores in the LCPFUAT trial at ages 4-5 years (−0.42 standard deviation, 95% confidence interval −0.71 to −0.13, n=184 out of 309 randomised, P=0.005) and in the LCPUFAP trial at age 16 years (−1.22, −2.29 to −0.14, n=17 out of 196 randomised, P=0.026). In both follow-ups, attrition was high (40.5% and 91%, respectively), however, which made the findings difficult to interpret.

## Discussion

### Principal findings

We found no evidence of improved cognitive ability, measured by differences in scores for GCSE mathematics at age 16 years, for nutrient enriched versus standard formula in infants born small for gestational age at term, or in preterm infants after discharge from hospital, in LCPUFA supplemented versus unsupplemented formula in term or preterm infants, or in high versus low iron follow-on formula in term infants. Upper 95% confidence limits excluded a moderate benefit of more than 0.27 standard deviation for any of the modified formulas (equivalent to about half a GCSE grade). Sensitivity analyses showed the robustness of these findings to covariate adjustment, methods of handling missing data, and standardisation reference distribution. Two trials where no cognitive effects were expected (sn-2 palmitate supplemented formula and nucleotide supplemented formula) showed no effects on academic performance, further demonstrating the robustness of the overall study methodology. Previous assessments of the randomised controlled trials included in our study, and findings from other randomised controlled trials of these modified formulas, are consistent with our findings of no evidence of cognitive benefit (supplement 1).^5^ ^6^ ^7^ ^11^ ^31^ ^32^ ^33^

We found weak evidence of reduced performance in secondary academic performance measures at age 11 years in children randomised to the LCPUFA supplemented formula. Why LCPUFA supplemented infant formula might adversely affect academic performance is unclear. The content of docosahexaenoic acid in human milk is variable and greatly influenced by maternal diet.^34^ This natural variability in breast milk makes the optimal dose of docosahexaenoic acid in infant formula uncertain. Furthermore, LCPUFAs derived from sources other than human breast milk, and in isolation from other components present in human breast milk,^35^ likely have different biological properties compared with ‎LCPUFAs naturally occurring in human breast milk. Given the potential associations between the source of LCPUFAs and adverse cognitive outcomes, long term follow-up of trials testing infant formulas from other sources of LCPUFAs is recommended, including use of innovative trial designs.^36^ The findings of potential harm associated with LCPUFA supplementation in this study are particularly important because of the recent mandate to add one type of LCPUFA, docosahexaenoic acid, to all infant and follow-on formulas in the European Union.^3^ Combined with previous evidence, our findings should prompt the reappraisal of such legislation, which is mainly based on estimated requirements, short term outcomes, and expert consensus. A mandate might have the potential for considerable harm and could also inhibit future research by limiting equipoise.

### Strengths and weaknesses

A key strength of our study was the high rate of follow-up: 86% compared with an average of 48% achieved for cognitive outcomes at ages 6-16 years in previous studies (supplement 1). Attrition, exacerbated by a lack of funding for long term studies, threatens the validity of study findings by introducing bias caused by factors associated with cognitive ability that can disproportionately occur in children lost to follow-up. Attrition also reduces statistical power, making it less likely that important differences are detected.^10^ High rates of follow-up in our study were possible because of retention of the participants’ identifiers, and legal, governance, and ethics approvals that enabled linkage between trial data and routinely collected schools data with exemption from the need for participant consent on the basis of public benefit. The practice of storing identifiers on paper and their destruction after a set time is frequently requested by ethics committees. These practices undoubtedly limit the value and public benefit of the data. Data retention policies should be re-examined to allow linkable identifiers to be collected and preserved.^37^ Preservation of linkable identifiers could facilitate the re-analysis of important trials through linkage to routinely collected administrative data and provide regulators with relevant policies based on long term outcomes.

A further strength of our study was the use of mandatory high stakes national school examinations (GCSEs) as the cognitive endpoint. GCSE scores have real world relevance to young people and their future. The primary outcome, performance at GCSE mathematics, is a strong predictor of future success in the labour market.^23^ In contrast with IQ scores, small changes in performance at GCSE examinations can lead to a difference in final grades, with research illustrating how falling just above or below key grade boundaries can have important consequences for academic progression.^24^ Also, GCSE scores are routinely collected, independently of the trial. This type of collection can maintain blinding of the outcome assessor, save time for the participants, and increase rates of follow-up.^10^ GCSEs are high stakes examinations for children, meaning that participants try their best. In contrast, research assessments such as IQ tests are low stakes for participants because nothing depends on the results. A broad range of literature on educational assessments has shown that these types of assessment can lead to minimal effort,^38^ potentially introducing substantial measurement error into the results.^39^ ^40^

Several limitations apply to this study. Firstly, analyses of academic performance were powered to only detect differences between groups greater than 0.32-0.43 standard deviation. Smaller differences are considered to lack clinical significance in research on infant formulas^41^ because trials conventionally focus on outcomes where small changes are unlikely to have any substantial long term consequences (eg, Bayley scale or IQ scores). Smaller effect sizes in academic performance in adolescence could have a substantial effect on public health at a population level because GCSE results predict earnings in adulthood.^26^ ^42^ ^43^ ^44^ ^45^ Extremely large trials would be needed, however, with high rates of long term follow-up to confirm or rule out an effect size smaller than 0.32-0.43 standard deviation. Such trials would be difficult to justify, given the consistent lack of evidence of benefit.

Secondly, the trials were conducted several decades ago; the composition of formulas and neonatal care have since changed. Nowadays, a larger number of sick and small preterm infants survive,^46^ ^47^ and these infants might have different sensitivities to the nutritional modifications investigated in this study. Although our analyses included babies born at 25 weeks, our results might not be generalisable to babies born as early as 23 weeks. The main changes in the composition of formulas over time are the widespread addition of LCPUFAs (with docosahexaenoic acid mandated in all European formulas since 2020^3^), a trend towards higher protein in preterm formulas and lower protein in term formulas, and the addition of prebiotics. These changes have become almost universal, making randomisation to earlier formula compositions unacceptable and thereby inhibiting future trials, even though the long term safety and efficacy of today’s standard formulas remain largely unexplored.

Finally, the cognitive benefits of the randomised formula modifications could have been diluted by unmeasured compensatory interventions in the control group over time, masking a true benefit of the intervention. None of the trials we investigated reported evidence of cognitive advantage for the modified formula group in early childhood (before dilution could have taken place), however. Because no evidence of benefit was found at multiple developmental points during childhood in the included studies and from meta-analyses that included other randomised controlled trials,^5^ ^6^ ^7^ no support for the promotion of these products for cognitive benefit currently exists.

### Conclusions

In summary, differences in academic performance between modified and standard formulas were consistent with differences measured in the original trials and in the external literature; that is, no benefit of the infant formula modifications on cognitive outcomes. We have shown that following up dormant clinical trials through data linkage without participants having to give their consent again is feasible and leads to higher follow-up rates compared with conventional follow-up methods. This approach, with linkage to school data and potentially to health databases, can be used to maximise the use of dormant trials to look at the remaining gaps in evidence about modification of formula feeds at critical stages of development. This study sets a precedent for other trials and cohorts to use linkage to administrative data to answer important questions about long term outcomes in children and young people.

**What is already known on this topic**

Infant formula is consumed globally by more than 60% of infants aged <6 months
Unlike other early interventions to support cognitive development, infant formula modifications are highly scalable; evidence that modifications of infant formula result in long term cognitive advantages is important for public health
The benefits for long term cognitive ability that have been claimed for nutrient enriched formula, long chain polyunsaturated fatty acid supplemented formula, and high iron follow-on formula are uncertain because of the high attrition rate in randomised controlled trials

**What this study adds**

Attrition was minimised by linking seven dormant infant formula trials to administrative school data with section 251 (NHS Act 2006) support, instead of consent
Primary outcome data (results of GCSE mathematics examinations at age 16) were available for 86% of participants in the eligible age range
No cognitive benefit, as measured by academic performance, was found for any of the modified formulas (nutrient enriched, long chain polyunsaturated fatty acid supplemented, iron fortified follow-on formula, sn-2 palmitate supplemented, and nucleotide supplemented), consistent with the original trials and the external literature

## Data Availability

Data are subject to data sharing agreements and are not publicly available. Data could be obtained on retrieving the relevant permissions from the Health Research Authority, Confidentiality Advisory Group, Department for Education, and UCL. All statistical code can be obtained here https://github.com/MaxVerfuerden/infant-formula-and-academic-performance

## Web extra

Extra material supplied by authors

Web appendix: Supplementary materials

## Notes

Contributors: MLV prepared the initial draft of the manuscript and protocol, and conducted the analyses. RG, JJ, and MF provided substantial intellectual input. AL and MF conducted the original trials and had access to the trial data and participant identifiers. All authors contributed to critical revisions of the manuscript and approved the final version for submission. MLV acts as guarantor of the manuscript. The corresponding author attests that all listed authors meet authorship criteria and that no others meeting the criteria were omitted.

Patient consent: The study was conducted with support under section 251 of the NHS Act 2006 instead of participant consent to avoid bias caused by non-response that would arise because of difficulties locating participants in adolescence and adulthood (Health Research Authority (HRA) Confidentiality Advisory Group (CAG): IRAS ID: 212148/17CAG0051).

The lead author (the manuscript’s guarantor) affirms that the manuscript is an honest, accurate, and transparent account of the study being reported; that no important aspects of the study have been omitted; and that any discrepancies from the study as planned (and, if relevant, registered) have been explained.

Dissemination to participants and related patient and public communities: The results of this study will be disseminated to scientific, practitioner, and lay audiences at conferences, through open access papers, and a doctoral thesis. A lay summary of the results will be made available online (on GitHub and the UCL study website) and will be promoted on social media.

Provenance and peer review: Not commissioned; externally peer reviewed.
