# Supplementary material for: Effect of nutritionally modified infant formula on academic performance: linkage of seven dormant randomised controlled trials to national education data
Source: BMJ. 2021 Nov 11;375:e065805. doi: 10.1136/bmj-2021-065805 (PMC8579423; doi:10.1136/bmj-2021-065805)
Supplement: Supplementary file 1 — Web appendix: Supplementary materials [file verm065805.ww.pdf]

Supplementary File

Supplement 1: Characteristics of systematic reviews of RCTs and reported pooled results for all outcomes grouped by type of formula modification and characteristics at birth 2

Overview of search terms and strategies for each modification 6

Supplement 2: Power calculations and primary outcomes for the original trials 14

Post-linkage delta estimation for primary outcome 15

Supplement 3: Macronutrient compositions of intervention and control formulas in the different trials 16

Supplement 4: Consistency of school outcomes with previous in-trial measurements. Pearson’s correlation between cognitive measures (all trials combined) 17

Supplement 5: Robust standard errors used in this study 17

Supplement 6: Baseline characteristics at randomisation, by trial 18

Supplement 7: Primary, Secondary and Sensitivity analyses 19

Supplement 1: Characteristics of systematic reviews of RCTs and reported pooled results for all outcomes grouped by type of formula modification and characteristics at birth

Population	Study	Intervention	Relative effect of intervention vs control (95% CI)	Age	RCTs	% of participants followed-up (n/randomised)	With data from
Preterm	Young 2016 (Systematic review of RCTs)	Nutrient-enriched post-discharge formula vs standard term formula	Weight (grams) MD 7.45 g lower (-141.84 to 126.93)	3-4 months post-term	6	91% (523/576)	Koo 2006 Litmanovitz 2004 Lucas 1992 Lucas 2001 Roggero 2011 Roggero 2012
			Weight (grams) MD 35.54 g higher (-113.71 to 184.78)	6 months post-term	7	89% (576/646)	Atkinson 1999 Koo 2006 Litmanovitz 2004 Lucas 1992 Lucas 2001 Roggero 2011 Roggero 2012
			Crown-heel length (mm) MD 2.45 mm taller (-2.01 to 6.9)	3-4 months post-term	6	91% (523/576)	Koo 2006 Litmanovitz 2004 Lucas 1992 Lucas 2001 Roggero 2011 Roggero 2012
			Crown-heel length (mm) MD 2.12 mm taller (-2.16 to 6.41)	6 months post-term	7	89% (576/646)	Atkinson 1999 Koo 2006 Litmanovitz 2004 Lucas 1992 Lucas 2001 Roggero 2011 Roggero 2012
			Head circumference (mm) MD 0.3 mm smaller (-2.86 to 2.26)	3-4 months post-term	6	91% (523/576)	Koo 2006 Litmanovitz 2004 Lucas 1992 Lucas 2001 Roggero 2011 Roggero 2012
			Head circumference (mm) MD 2.28 mm larger (-0.28 to 4.83)	6 months post-term	7	89% (576/646)	Atkinson 1999 Koo 2006 Litmanovitz 2004 Lucas 1992 Lucas 2001 Roggero 2011 Roggero 2012
			Bayley Scales of Infant Development II: Mental Development Index MD 0.9 higher (-3.24 to 5.04)	18 months	1	81% (184/228)	Lucas 2001

	Embleton RCT 10 year follow up*		Wechsler Intelligence Scale for Children III full-scale IQ	10 years	1	71% (92/129)	Embleton 2021
SGA Term	Castanys-Muñoz 2017 (Systematic review that includes 2 RCTs, both reported in Singhal 2010)	Nutrient-enriched formula vs standard term formula	Crown-heel length (cm) MD 1.1 cm taller (0.38 to 1.79)	9 months	1	83% (247/299)	Singhal 2010
			Crown-heel length (cm) MD 1 cm taller (0.25 to 1.83)	18 months	1	80% (240/299)	Singhal 2010
			Head circumference (mm) MD 0.5 wider (0.1 to 0.9)	9 months	1	83% (247/299)	Singhal 2010
			Head circumference (mm) MD 0.6 wider (0.2 to 1.1)	18 months	1	80% (240/299)	Singhal 2010
			Knobloch, Pasamanick, and Sherrard's developmental screening inventory MD 2.5 lower (-4.6 to -0.4)	9 months	1	82% (246/299)	Singhal 2010
			Bayley Scales of Infant Development II: Mental Development Index MD 1.5 lower (-5.1 to 2.0)	18 months	1	79% (235/299)	Singhal 2010
			Per cent body fat MD 38.7% higher (9.6 to 67.8%)	6-8 years	1	51% (153/299)	Singhal 2010
			Per cent body fat MD 18% higher (0.3 to 36%)	6-8 years	1	37% (90/246)	Singhal 2010
			Blood pressure (diastolic) MD 3.5 mm Hg higher (0.7 to 6.2 mm Hg)	6-8 years	1	51% (153/299)	Singhal 2010
Preterm	Moon 2016 (Systematic review of RCTs)	LCPUFA-supplemented infant formula vs standard preterm formula	Bayley Scales of Infant Development II: Mental Development Index MD 0.96 higher (-1.42 to 3.34)	12 months post-term	4	58% (364/633)	Carlson 1992<br>Fang 2005<br>O'Connor 2001<br>van Wezel 2002
			Bayley Scales of Infant Development II: Mental Development Index MD 0.7 lower (-2.63 to 1.23)	18 months post-term	3	62% (494/795)	Clandinin 2005<br>Fewtrell 2002<br>Fewtrell 2004
			Weight (kg) MD 0.10 lower (-0.31 to 0.12)	12 months post-term	4	43% (271/636)	Carlson 1996<br>Clandinin 2005<br>Groh-Wargo 2005<br>Vanderhoof 1999
			Length (cm) MD 0.25 smaller (≥0.33 to 0.84)	12 months post-term	4	43% (271/636)	Carlson 1996<br>Clandinin 2005<br>Groh-Wargo 2005<br>Vanderhoof 1999
	Verfuerden 2020 (Systematic review of RCTs)		WASI IQ MD 7.71 lower (-24.63 to 9.22)	10-17 years	2	29% (124/434)	Fewtrell 2002<br>Fewtrell 2004
Term	Jasani 2017 (Systematic	LCPUFA-supplemented infant	Visual acuity MD 0.01 lower (-0.12 to 0.11)	12 months	3	44% (256/577)	Auestad 1997<br>Auestad 2001

	review of RCTs)	formula vs standard term formula					Carlson 1996
			Sweep VEP acuity MD 0.15 lower (-0.17 to -0.13)	12 months	3	69% (244/352)	Birch 1998<br>Birch 2005<br>Birch 2010
			Bayley Scales of Infant Development II: Mental Development Index MD 0.06 higher (-2.01 to 2.14)	18 months	4	76% (661/873)	Birch 1998<br>Birch 2010<br>Bouwstra 2005<br>Lucas 1999
			Weight z-score MD 0.23 SD lower (-0.40 to -0.06)	12 months	5	60% (521/871)	Agostoni 1995<br>Auestad 1997<br>Auestad 2001<br>Birch 2005<br>Birch 2010
			Length z-score MD 0.04 lower (-0.19 to 0.11)	12 months	5	60% (521/871)	Agostoni 1995<br>Auestad 1997<br>Auestad 2001<br>Birch 2005<br>Birch 2010
			Head circumference MD 0.13 smaller (-0.32 to 0.05)	12 months	4	51% (464/911)	Auestad 2001<br>Bouwstra 2005<br>Makrides 1999<br>Morris 2000
	Verfuerden 2020 (Systematic review of RCTs)		WPPSI IQ MD 0.04 lower (-5.94 to 5.85)	4-6 years	4	62% in trials where original number randomised is known (578/939)	Lucas unpublished<br>Willats 2013<br>Birch 2007<br>Colombo 2013
Term	Santiago Chile Preventive Trial (RCT)	Iron-fortified follow-on formula vs formula with no added iron in healthy term infants	Weight for age z-score any vs none MD 0.08 lower (-0.19 to 0.03)	12 months	1	92% (1657/1798)	Lozoff 2003
			Length for age z-score any vs none MD 0.12 lower (-0.22 to -0.02)	12 months	1	92% (1657/1798)	Lozoff 2003
			Bayley Scales of Infant Development I: Mental Development Index MD 0.7 lower (-2.63 to 1.23)	12 months	1	92% (1613/1798)	Lozoff 2003
		Iron-fortified follow-on formula (12.7 mg/L) vs low iron (2.3 mg/L) formula in healthy term infants	Bayley Scales of Infant Development I: Mental Development Index MD 1.5 higher (-0.7 to 3.71)	12 months	3	42% (473/1123)	Lozoff 2012
			IQ, WISC: 0.13 lower (-0.25 to -0.01)	10 years	1	42% (473/1123)	Lozoff 2012
			Arithmetic achievement, WRAT 2.4 lower (-4.5 to -0.3)	16 years	1	36% (403/1123)	Gahagan 2019

	Morley 1999 (RCT)	Iron-fortified follow-on formula (12mg/100ml) vs low iron (0.9 mg/100ml) formula in healthy term infants	Bayley Scales of Infant Development II: Mental Development Index MD 0.6 lower (-3.40 to 2.20)	18 months	1	82% (268/327)	Morley 1999
			Head circumference (cm) MD 0.1 cm wider (-0.27 to 0.47)	18 months	1	82% (268/327)	Morley 1999
			Length (cm) MD 0 cm taller (≥0.71 to 0.71)	18 months	1	82% (268/327)	Morley 1999
		Iron-fortified follow-on formula (12mg/100ml) vs cows's milk (approx. 0.05mg/l) in healthy term infants	Bayley Scales of Infant Development II: Mental Development Index MD 2.3 lower (-4.87 to 0.27)	18 months	1	89% (293/328)	Morley 1999
			Head circumference (cm) MD 0.1 cm wider (-0.27 to 0.47)	18 months	1	89% (293/328)	Morley 1999
			Length (cm) MD 0.3 cm smaller (≥1.01 to 0.41)	18 months	1	89% (293/328)	Morley 1999
	Iglesias Vázquez 2019 (RCT)	Iron-fortified follow-on formula (1.2 mg/100ml) vs low iron (0.4 mg/100ml) formula between 6-9 months in healthy term infants	Bayley Scales of Infant Development II: Mental Development Index MD 3.3 points higher in intervention group (p=0.217)<br>Head circumference (cm) MD 0.8 cm wider in intervention group (p=0.012) *difference was already statistically significant at randomisation (6 months)<br>Length (cm) MD 1.8 cm taller in intervention group (p=0.007) *difference was already statistically significant at randomisation (6 months)	12 months	1	94% (133/142)<br>* 28 infants in control group and 105 in intervention group	Iglesias Vázquez 2019
Term	No studies with cognitive ability outcomes found						
Tern	No studies with cognitive ability outcomes found						

CI = Confidence Interval, MD = Mean difference, RCT= Randomised Controlled Trial, IQ= Intelligence Quotient, SGA= Small for gestational age; *added post-hoc

Overview of search terms and strategies for each modification

The search terms to identify systematic reviews in MEDLINE® and EMBASE were adapted from the Scottish Intercollegiate Guidelines Network.

MEDLINE® terms for cognitive ability
("Cognition"[MeSH Terms] OR "Child Development"[Mesh] OR "Intelligence"[Mesh] OR "Brain/growth and development"[Mesh] OR "Cognition"[TIAB] OR "Child Development"[TIAB] OR "Intelligence"[TIAB] OR "Cognitive function"[TIAB] OR "Learning"[TIAB] OR "Cognitive test"[TIAB] OR "Brain"[TIAB] OR "neuro*development"[TIAB] OR "educational status"[MeSH Terms] OR "education*"[TIAB] OR "educational status"[TIAB] OR "schools"[MeSH Terms] OR "schools"[TIAB] OR "school"[TIAB] OR attainment[TIAB] OR "Bayley"[TIAB])

MEDLINE® terms for infant nutrition
AND ("Infant Formula"[Mesh] OR "Infant Food"[Mesh] OR "Infant Nutritional Physiological Phenomena"[Mesh] OR "Food, Fortified"[MAJR] OR "Nutritional Support"[TIAB] OR "follow*on*formula"[TIAB] OR "supplementation"[TIAB])

MEDLINE® terms for study population
(("infant"[MeSH Terms] OR "child"[MeSH Terms] OR "adolescent"[MeSH Terms]) OR (infant[TIAB] OR child[TIAB] OR adolescent[TIAB]))

MEDLINE® terms for systematic review of RCTs
(Review[ptyp] OR ((systematic review[ti] OR meta-analysis[pt] OR meta-analysis[ti] OR systematic literature review[ti] OR this systematic review[tw] OR pooling project[tw] OR (systematic review[tiab] AND review[pt]) OR meta synthesis[ti] OR (meta analysis[ti] OR meta analyses[ti] OR meta analyse[ti] OR meta analysed[ti] OR meta analyser[ti] OR meta analyses[ti] OR meta analysing[ti] OR meta analysis[ti] OR meta analysis,[ti] OR meta analysisdagger[ti] OR meta analysis of[ti] OR meta analyst[ti] OR meta analysticians[ti] OR meta analysts[ti] OR meta analysys[ti] OR meta analytic[ti] OR meta analytical[ti] OR meta analytically[ti] OR meta analyze[ti] OR meta analyzed[ti] OR meta analyzes[ti] OR meta analyzing[ti]) OR integrative review[tw] OR integrative research review[tw] OR rapid review[tw] OR umbrella review[tw] OR consensus development conference[pt] OR practice guideline[pt] OR drug class reviews[ti] OR "Cochrane Database Syst Rev"[Journal] OR "ACP J Club"[Journal] OR "Health Technol Assess"[Journal] OR "Evid Rep Technol Assess (Summ)"[Journal] OR "JBI Database System Rev Implement Rep"[Journal]) OR (clinical guideline[tw] AND management[tw]) OR ((evidence based[ti] OR "evidence-based medicine"[MeSH Terms] OR (best practice[ti] OR best practices[ti]) OR evidence synthesis[tiab]) AND (review[pt] OR diseases category[mh] OR "behaviour and behaviour mechanisms"[MeSH Terms] OR "therapeutics"[MeSH Terms] OR evaluation studies[pt] OR validation studies[pt] OR guideline[pt] OR pmcbook[All Fields])) OR ((systematic[tw] OR systematically[tw] OR critical[tiab] OR study selection[tw] OR (predetermined[tw] OR inclusion[tw] AND (criteri[tw] OR criteria[tw] OR criteria'[tw] OR criteria'double[tw] OR criteria's[tw] OR criteria'srandomized[tw] OR criteria1[tw] OR criteria2[tw] OR criteriaadult[tw] OR criteriaall[tw] OR criteriaare[tw] OR criteriabased[tw] OR criteriadisulfiram[tw] OR criteriae[tw] OR criteriaeditorials[tw] OR criteriaen[tw] OR criteriaenglish[tw] OR criteriaexclusion[tw] OR criteriafor[tw] OR criteriafora[tw] OR criteriaheath[tw] OR criteriai[tw] OR criteriaincluded[tw] OR criteriaithe[tw] OR criterial[tw] OR criterialism[tw] OR criteriality[tw] OR criteriall[tw] OR criterially[tw] OR criterials[tw] OR criterian[tw] OR criteriaof[tw] OR criteriar[tw] OR criteriarandomised[tw] OR criteriarpar[tw] OR criterias[tw] OR criteriasof[tw] OR criteriastudies[tw] OR criteriasystematic[tw] OR criteriathe[tw] OR criteriation[tw] OR criteriatrade[tw] OR criteriaum[tw] OR criteriawerehaving[tw] OR criteric[tw] OR criterid[tw] OR criterien[tw] OR criteries[tw] OR criteriia[tw] OR criterin[tw] OR criterio[tw] OR criterioe[tw] OR criteriologic[tw] OR criteriological[tw] OR criteriology[tw] OR criterion[tw] OR criterion'[tw] OR criterion's[tw] OR criterional[tw] OR criterionby[tw] OR criterionis[tw] OR criterionoriented[tw] OR criterions[tw] OR criterior[tw] OR criteriors[tw] OR criterios[tw] OR criteriosa[tw] OR criteriosamente[tw] OR criterioso[tw] OR criterious[tw] OR criteris[tw] OR criterita[tw] OR criterium[tw] OR criterium'[tw] OR criteriums[tw] OR criterization[tw])) OR (exclusion criteria[tw] OR exclusion criterias[tw] OR exclusion criterion[tw] OR exclusion criterions[tw] OR exclusion criterium[tw]) OR main outcome measures[tw] OR standard of care[tw] OR standards of care[tw]) AND (survey[tiab] OR surveys[tiab] OR (overview[tw] OR overview'[tw] OR overview's[tw] OR overview2[tw] OR overviewed[tw] OR overviewer[tw] OR overviewers[tw] OR overviewes[tw] OR overviewing[tw] OR overviewn[tw] OR overviewon[tw] OR overviewpredictive[tw] OR overviewprognostic[tw] OR overviews[tw] OR overviews'[tw] OR overviews"[tw] OR overviewstudy[tw]) OR review[tiab] OR reviews[tiab] OR (search[tw] OR search'[tw] OR search's[tw] OR search010[tw] OR search013[tw] OR search1[tw] OR search5[tw] OR searchability[tw] OR searchable[tw] OR searchableby[tw] OR searchall[tw] OR searchamerica[tw] OR searchand[tw] OR searchback[tw] OR searchbreast[tw] OR searchcoil[tw] OR searchcompare[tw] OR searchdb[tw] OR searchdisease[tw] OR searchdogs[tw] OR searche[tw] OR searcheable[tw] OR searched[tw] OR searched'[tw] OR searched19[tw] OR searchedfor[tw] OR searchedmedline[tw] OR searchedwas[tw] OR searcheed[tw] OR searchen[tw] OR searcher[tw] OR searcher'[tw] OR searcher's[tw] OR searchers[tw] OR searchers'[tw] OR searches[tw] OR searches'[tw] OR searchescohorts[tw] OR searchfor[tw] OR searchform[tw] OR searchgenes[tw] OR searchgtr[tw] OR searchgui[tw] OR searchhes[tw] OR searchin[tw] OR searchin'[tw] OR searching[tw] OR searching'[tw] OR searchinger[tw] OR searchingfor[tw] OR searchingly[tw] OR searchings[tw] OR searchlight[tw] OR searchlight'[tw] OR searchlights[tw] OR searchlighttrade[tw] OR searchline[tw]

OR searchlite[tw] OR searchlyte[tw] OR searchmedica[tw] OR searchomyces[tw] OR searchpageeng[tw] OR searchpath[tw] OR searchpaths[tw] OR searchpatterns[tw] OR searchpattool[tw] OR searchpks[tw] OR searchproj[tw] OR searchresult[tw] OR searchs[tw] OR searchshowed[tw] OR searchsmallrna[tw] OR searchsnp[tw] OR searchtesv[tw] OR searchtm[tw] OR searchtrade[tw] OR searchtxt[tw] OR searchtype[tw] OR searchwise[tw] OR searchxlinks[tw]) OR handsearch[tw] OR analysis[ti] OR critique[tiab] OR appraisal[tw] OR (reduction[tw] AND ("risk"[MeSH Terms] OR risk[tw]) AND (("death"[MeSH Terms] OR "death"[All Fields]) OR ("recurrence"[MeSH Terms] OR "recurrence"[All Fields])))) AND (literature[tiab] OR articles[tiab] OR publications[tiab] OR publication[tiab] OR bibliography[tiab] OR bibliographies[tiab] OR published[tiab] OR pooled data[tw] OR unpublished[tw] OR citation[tw] OR citations[tw] OR database[tiab] OR internet[tiab] OR textbooks[tiab] OR references[tw] OR scales[tw] OR papers[tw] OR datasets[tw] OR trials[tiab] OR (meta analyis[tw] OR meta analyisis[tw] OR meta analysable[tw] OR meta analysas[tw] OR meta analyse[tw] OR meta analysed[tw] OR meta analysei[tw] OR meta analysen[tw] OR meta analyser[tw] OR meta analysers[tw] OR meta analyses[tw] OR meta analysescohort[tw] OR meta analysespublication[tw] OR meta analysestype[tw] OR meta analysi[tw] OR meta analysia[tw] OR meta analysic[tw] OR meta analysing[tw] OR meta analysis[tw] OR meta analysis's[tw] OR meta analysis,[tw] OR meta analysis12[tw] OR meta analysis2[tw] OR meta analysisbone[tw] OR meta analysisdagger[tw] OR meta analysises[tw] OR meta analysisevaluating[tw] OR meta analysisif[tw] OR meta analysisindicated[tw] OR meta analysisintroduction[tw] OR meta analysisjr[tw] OR meta analysismethods[tw] OR meta analysismoderate[tw] OR meta analysisof[tw] OR meta analysistrade[tw] OR meta analysisv[tw] OR meta analysisxs[tw] OR meta analysized[tw] OR meta analyst[tw] OR meta analysticians[tw] OR meta analysts[tw] OR meta analysys[tw] OR meta analytic[tw] OR meta analytical[tw] OR meta analytically[tw] OR meta analytics[tw] OR meta analyzable[tw] OR meta analyze[tw] OR meta analyzed[tw] OR meta analyzes[tw] OR meta analyzing[tw]) OR (clinical[tiab] AND studies[tiab]) OR "treatment outcome"[MeSH Terms] OR treatment outcome[tw] OR pmcbook[All Fields])) NOT (letter[pt] OR newspaper article[pt])) OR (((randomized controlled trial[pt]) OR (controlled clinical trial[pt]) OR (randomized[tiab] OR randomised[tiab]) OR (placebo[tiab]) OR (drug therapy[sh]) OR (randomly[tiab]) OR (trial[tiab]) OR (groups[tiab])) NOT (animals[mh] NOT humans[mh])))

Enriched post-discharge formula for preterm infants

Embase

#	Searches
1	exp Meta Analysis/
2	((meta adj analy$) or metaanalys$).tw.
3	(systematic adj (review$1 or overview$1)).tw.
4	or/1-3
5	cancerlit.ab.
6	cochrane.ab.
7	embase.ab.
8	(psychlit or psyclit).ab.
9	(psychinfo or psycinfo).ab.
10	(cinahl or cinhal).ab.
11	science citation index.ab.
12	bids.ab.
13	or/5-12
14	reference lists.ab.
15	bibliograph$.ab.
16	hand-search$.ab.
17	manual search$.ab.
18	relevant journals.ab.
19	or/14-18
20	data extraction.ab.
21	selection criteria.ab.
22	20 or 21
23	review.pt.
24	22 and 23
25	letter.pt.
26	editorial.pt.
27	animal/
28	human/
29	27 not (27 and 28)
30	or/25-26,29
31	4 or 13 or 19 or 24
32	31 not 30
33	infant nutrition.tw. or exp infant nutrition/ or nutritional support.tw. or exp nutritional support/ or infant$food.tw. or infant$diet.tw. or baby$food.tw. or baby$diet.tw. or exp baby food/ or exp artificial milk/

34	exp cognition assessment/ or Cognition.tw. or exp cognition/ or exp social cognition/ or IQ.tw. or intelligence.tw. or exp Wechsler adult intelligence scale/ or exp intelligence quotient/ or exp Wechsler intelligence scale/ or exp "Wechsler preschool and primary scale of intelligence"/ or exp intelligence/ or exp Wechsler intelligence scale for children/ or exp intelligence test/ or exp emotional intelligence/ or Intelligence.mp. or exp Stanford-Binet Intelligence Scale/ or (neurodevelopment or cognitive).tw. or child$development.tw. or exp child development/ or exp neuropsychological test/ or exp child development/ or exp "Bayley Scales of Infant Development"/ or exp mental development/ or bayley.tw. or exp reading/ or exp school/ or exp learning disorder/ or exp educational status/ or exp education/ or exp achievement/ or exp academic achievement/ or school attainment.tw.
35	(child$ or infant$ or baby or babies or adolescent$ or teenager$).tw.
36	dietary proteins.tw. or exp protein intake/ or energy intake.tw. or exp caloric intake/ or enriched formula.tw. or exp enteric feeding/ or exp diet supplementation/ or preterm$formula.tw.
37	exp premature labor/ or exp prematurity/ or exp low birth weight/ or preterm$.tw. or premie$.tw. or low gestational age.tw. or low birthweight.tw. or exp low birth weight/ or small for gestational age.tw. or smallfordate$.tw. or exp small for date infant/
38	exp hospital discharge/ or post$discharge.tw. or after$discharge.tw. or following$discharge.tw. or hospital$discharge.tw.
39	32 and 33 and 34 and 35 and 36 and 37 and 38

MEDLINE
MEDLINE terms for cognitive ability, study population, premature infants, systematic review and infant nutrition (see above) plus:

Dietary Proteins"[Mesh] OR "Energy Intake"[Mesh] OR "Nutritional Support"[Mesh] OR "Dietary Proteins"[TIAB] OR "Energy Intake"[TIAB] OR "enriched formula"[TIAB] OR "supplemented formula"[TIAB] OR "Nutrient*enriched"[TIAB] OR "preterm*formula"[TIAB]) AND (("Hospital*"[TIAB] OR Discharge[TIAB]) AND ("after"[TIAB] OR "post"[TIAB] OR "following"[TIAB]))

LCPUFA-enriched infant formula for preterm infants

Embase

#	Searches
1	exp Meta Analysis/
2	((meta adj analy$) or metaanalys$).tw.
3	(systematic adj (review$1 or overview$1)).tw.
4	or/1-3
5	cancerlit.ab.
6	cochrane.ab.
7	embase.ab.
8	(psychlit or psyclit).ab.
9	(psychinfo or psycinfo).ab.
10	(cinahl or cinhal).ab.
11	science citation index.ab.
12	bids.ab.
13	or/5-12
14	reference lists.ab.
15	bibliograph$.ab.
16	hand-search$.ab.
17	manual search$.ab.
18	relevant journals.ab.
19	or/14-18
20	data extraction.ab.
21	selection criteria.ab.
22	20 or 21
23	review.pt.
24	22 and 23
25	letter.pt.
26	editorial.pt.
27	animal/
28	human/
29	27 not (27 and 28) (89)
30	or/25-26,29
31	4 or 13 or 19 or 24

32	31 not 30
33	infant nutrition.tw. or exp infant nutrition/ or nutritional support.tw. or exp nutritional support/ or infant$food.tw. or infant$diet.tw. or baby$food.tw. or baby$diet.tw. or exp baby food/ or exp artificial milk/
34	exp cognition assessment/ or Cognition.tw. or exp cognition/ or exp social cognition/ or IQ.tw. or intelligence.tw. or exp Wechsler adult intelligence scale/ or exp intelligence quotient/ or exp Wechsler intelligence scale/ or exp "Wechsler preschool and primary scale of intelligence"/ or exp intelligence/ or exp Wechsler intelligence scale for children/ or exp intelligence test/ or exp emotional intelligence/ or Intelligence.mp. or exp Stanford-Binet Intelligence Scale/ or (neurodevelopment or cognitive).tw. or child$development.tw. or exp child development/ or exp neuropsychological test/ or exp child development/ or exp "Bayley Scales of Infant Development"/ or exp mental development/ or bayley.tw. or exp reading/ or exp school/ or exp learning disorder/ or exp educational status/ or exp education/ or exp achievement/ or exp academic achievement/ or school attainment.tw.
35	(child$ or infant$ or baby or babies or adolescent$ or teenager$).tw.
36	exp fatty acids, omega-3/ or fatty acids, essential/ or Dietary Fats, Unsaturated/ or linolenic acids/ or exp fish oils/ or (n 3 fatty acid$ or omega 3).tw. or docosahexa?noic.tw,hw,rw. or eicosapenta?noic.tw,hw,rw. or alpha linolenic.tw,hw,rw. or (linolenate or cervonic or timnodonic).tw,hw,rw. or menhaden oil$.tw,hw,rw. or (mediterranean adj diet$).tw. or ((flax or flaxseed or flax seed or linseed or rape seed or rapeseed or canola or soy or soybean or walnut or mustard seed) adj2 oil$).tw. or (walnut$ or butternut$ or soybean$ or pumpkin seed$).tw. or (fish adj2 oil$).tw. or (cod liver oil$ or marine oil$ or marine fat$).tw. or (salmon or mackerel or herring or tuna or halibut or seal or seaweed or anchov$).tw. or (fish consumption or fish intake or (fish adj2 diet$)).tw. or diet$ fatty acid$.tw. or borage oil$.tw.
37	exp premature labor/ or exp prematurity/ or exp low birth weight/ or preterm$.tw. or premie$.tw. or low gestational age.tw. or low birthweight.tw. or exp low birth weight/ or small for gestational age.tw. or smallfordate$.tw. or exp small for date infant/
38	32 and 33 and 34 and 35 and 36 and 37

MEDLINE
MEDLINE terms for cognitive ability, study population, premature infants, systematic review and infant nutrition (see above) plus:

("Fatty Acids, Unsaturated"[Mesh] OR "Arachidonic Acids"[Mesh] OR "Docosahexaenoic Acids"[Mesh] OR "LCPUFA"[TIAB] OR "PUFA"[TIAB] OR "Borage Oil"[TIAB] OR "Fish Oil"[TIAB] OR "Arachidonic Acids"[TIAB] OR "Docosahexaenoic Acids"[TIAB] OR "fatty acid"[TIAB] OR "omega 3"[TIAB])

LCPUFA-enriched infant formula for term infants
Embase

#	Searches
1	exp Meta Analysis/
2	((meta adj analy$) or metaanalys$).tw.
3	(systematic adj (review$1 or overview$1)).tw.
4	or/1-3
5	cancerlit.ab.
6	cochrane.ab.
7	embase.ab.
8	(psychlit or psyclit).ab.
9	(psychinfo or psycinfo).ab.
10	(cinahl or cinhal).ab.
11	science citation index.ab.
12	bids.ab.
13	or/5-12
14	reference lists.ab.
15	bibliograph$.ab.
16	hand-search$.ab.
17	manual search$.ab.
18	relevant journals.ab.
19	or/14-18
20	data extraction.ab.
21	selection criteria.ab.
22	20 or 21
23	review.pt.
24	22 and 23
25	letter.pt.
26	editorial.pt.
27	animal/
28	human/

29	27 not (27 and 28)
30	or/25-26,29
31	4 or 13 or 19 or 24
32	31 not 30
33	infant nutrition.tw. or exp infant nutrition/ or nutritional support.tw. or exp nutritional support/ or infant$food.tw. or infant$diet.tw. or baby$food.tw. or baby$diet.tw. or exp baby food/ or exp artificial milk/
34	exp cognition assessment/ or Cognition.tw. or exp cognition/ or exp social cognition/ or IQ.tw. or intelligence.tw. or exp Wechsler adult intelligence scale/ or exp intelligence quotient/ or exp Wechsler intelligence scale/ or exp "Wechsler preschool and primary scale of intelligence"/ or exp intelligence/ or exp Wechsler intelligence scale for children/ or exp intelligence test/ or exp emotional intelligence/ or Intelligence.mp. or exp Stanford-Binet Intelligence Scale/ or (neurodevelopment or cognitive).tw. or child$development.tw. or exp child development/ or exp neuropsychological test/ or exp child development/ or exp "Bayley Scales of Infant Development"/ or exp mental development/ or bayley.tw. or exp reading/ or exp school/ or exp learning disorder/ or exp educational status/ or exp education/ or exp achievement/ or exp academic achievement/ or school attainment.tw.
35	(child$ or infant$ or baby or babies or adolescent$ or teenager$).tw.
36	exp fatty acids, omega-3/ or fatty acids, essential/ or Dietary Fats, Unsaturated/ or linolenic acids/ or exp fish oils/ or (n 3 fatty acid$ or omega 3).tw. or docosahexa?noic.tw,hw,rw. or eicosapenta?noic.tw,hw,rw. or alpha linolenic.tw,hw,rw. or (linolenate or cervonic or timnodonic).tw,hw,rw. or menhaden oil$.tw,hw,rw. or (mediterranean adj diet$).tw. or ((flax or flaxseed or flax seed or linseed or rape seed or rapeseed or canola or soy or soybean or walnut or mustard seed) adj2 oil$).tw. or (walnut$ or butternut$ or soybean$ or pumpkin seed$).tw. or (fish adj2 oil$).tw. or (cod liver oil$ or marine oil$ or marine fat$).tw. or (salmon or mackerel or herring or tuna or halibut or seal or seaweed or anchov$).tw. or (fish consumption or fish intake or (fish adj2 diet$)).tw. or diet$ fatty acid$.tw. or borage oil$.tw.
37	32 and 33 and 34 and 35 and 36

MEDLINE terms for cognitive ability, study population, systematic review and infant nutrition (see above) plus:

("Fatty Acids, Unsaturated"[Mesh] OR "Arachidonic Acids"[Mesh] OR "Docosahexaenoic Acids"[Mesh] OR "LCPUFA"[TIAB] OR "PUFA"[TIAB] OR "Borage Oil"[TIAB] OR "Fish Oil"[TIAB] OR "Arachidonic Acids"[TIAB] OR "Docosahexaenoic Acids"[TIAB] OR "fatty acid"[TIAB] OR "omega 3"[TIAB])

Iron-fortified infant formula

Embase

#	Searches
1	exp Meta Analysis/
2	((meta adj analy$) or metaanalys$).tw.
3	(systematic adj (review$1 or overview$1)).tw.
4	or/1-3
5	cancerlit.ab.
6	cochrane.ab.
7	embase.ab.
8	(psychlit or psyclit).ab.
9	(psychinfo or psycinfo).ab.
10	(cinahl or cinhal).ab.
11	science citation index.ab.
12	bids.ab.
13	or/5-12
14	reference lists.ab.
15	bibliograph$.ab.
16	hand-search$.ab.
17	manual search$.ab.
18	relevant journals.ab.
19	or/14-18
20	data extraction.ab.
21	selection criteria.ab.
22	20 or 21
23	review.pt.
24	22 and 23
25	letter.pt.
26	editorial.pt.
27	animal/
28	human/
29	27 not (27 and 28)

30	or/25-26,29
31	4 or 13 or 19 or 24
32	31 not 30
33	infant nutrition.tw. or exp infant nutrition/ or nutritional support.tw. or exp nutritional support/ or infant$food.tw. or infant$diet.tw. or baby$food.tw. or baby$diet.tw. or exp baby food/ or exp artificial milk/
34	exp cognition assessment/ or Cognition.tw. or exp cognition/ or exp social cognition/ or IQ.tw. or intelligence.tw. or exp Wechsler adult intelligence scale/ or exp intelligence quotient/ or exp Wechsler intelligence scale/ or exp "Wechsler preschool and primary scale of intelligence"/ or exp intelligence/ or exp Wechsler intelligence scale for children/ or exp intelligence test/ or exp emotional intelligence/ or Intelligence.mp. or exp Stanford-Binet Intelligence Scale/ or (neurodevelopment or cognitive).tw. or child$development.tw. or exp child development/ or exp neuropsychological test/ or exp child development/ or exp "Bayley Scales of Infant Development"/ or exp mental development/ or bayley.tw. or exp reading/ or exp school/ or exp learning disorder/ or exp educational status/ or exp education/ or exp achievement/ or exp academic achievement/ or school attainment.tw.
35	(child$ or infant$ or baby or babies or adolescent$ or teenager$).tw.
36	exp iron metabolism/ or exp iron/ or exp iron blood level/ or exp iron deficiency/ or exp iron derivative/ or exp iron deficiency anemia/ or iron complex/ or exp iron intake/ or exp iron depletion/ or iron.tw. or ferritin$.tw. or exp ferritin blood level/ or exp ferritin/ or Hemoglobin$.tw.
37	32 and 33 and 34 and 35 and 36

MEDLINE

MEDLINE terms for cognitive ability, systematic review and infant nutrition (see above) plus:

("iron"[MeSH Terms] OR "Anemia, Iron-Deficiency"[Mesh] OR "Ferritins"[Mesh] OR "Iron, Dietary"[Mesh] OR "Hemoglobins"[Mesh] OR "iron"[TIAB] OR "Iron*Deficiency"[TIAB] OR "Ferritins"[TIAB] OR "Hemoglobins"[TIAB])

Sn-2 Palmitate

Embase

#	Searches	Results
1	exp Meta Analysis/	144648
2	((meta adj analy$) or metaanalys$).tw.	168465
3	(systematic adj (review$1 or overview$1)).tw.	148021
4	or/1-3	291254
5	cancerlit.ab.	740
6	cochrane.ab.	79808
7	embase.ab.	82418
8	(psychlit or psyclit).ab.	996
9	(psychinfo or psycinfo).ab.	20842
10	(cinahl or cinhal).ab.	24293
11	science citation index.ab.	3312
12	bids.ab.	571
13	or/5-12	128593
14	reference lists.ab.	17025
15	bibliograph$.ab.	19151
16	hand-search$.ab.	7008
17	manual search$.ab.	4406
18	relevant journals.ab.	1313
19	or/14-18	43993
20	data extraction.ab.	19994
21	selection criteria.ab.	33126
22	20 or 21	50993
23	review.pt.	2353244
24	22 and 23	25406
25	letter.pt.	1013577
26	editorial.pt.	562504
27	animal/	1840342
28	human/	19481735
29	27 not (27 and 28)	79485
30	or/25-26,29	1655532
31	4 or 13 or 19 or 24	345681
32	31 not 30	336477
33	infant nutrition.tw. or exp infant nutrition/ or nutritional support.tw. or exp nutritional support/ or infant$food.tw. or infant$diet.tw. or baby$food.tw. or baby$diet.tw. or exp baby food/ or exp artificial milk/	101863

34	exp cognition assessment/ or Cognition.tw. or exp cognition/ or exp social cognition/ or IQ.tw. or intelligence.tw. or exp Wechsler adult intelligence scale/ or exp intelligence quotient/ or exp Wechsler intelligence scale/ or exp "Wechsler preschool and primary scale of intelligence"/ or exp intelligence/ or exp Wechsler intelligence scale for children/ or exp intelligence test/ or exp emotional intelligence/ or Intelligence.mp. or exp Stanford-Binet Intelligence Scale/ or (neurodevelopment or cognitive).tw. or child$development.tw. or exp child development/ or exp neuropsychological test/ or exp child development/ or exp "Bayley Scales of Infant Development"/ or exp mental development/ or bayley.tw. or exp reading/ or exp school/ or exp learning disorder/ or exp educational status/ or exp education/ or exp achievement/ or exp academic achievement/ or school attainment.tw.	3302096
35	(child$ or infant$ or baby or babies or adolescent$ or teenager$).tw.	1963467
36	palmitate.tw. or exp palmitic acid/ or palmitic acid.tw. or sn-2 palmitate.tw. or triglyceride.tw. or exp triacylglycerol/	214049
37	crossover-procedure/ or double-blind procedure/ or randomized controlled trial/ or single-blind procedure/ or (random* or factorial* or crossover* or cross over* or placebo* or (doubl* adj blind*) or (singl* adj blind*) or assign* or allocat* or volunteer*).tw.	1975974
38	32 or 37	2180399
39	33 and 34 and 35 and 36 and 38	13

MEDLINE®

MEDLINE terms for cognitive ability, study population, and infant nutrition (see above) and …

("Palmitic Acids"[Mesh] OR "Triglycerides"[Mesh] OR "Beta*palmitate"[TIAB] OR "sn-2 palmitate"[TIAB] OR "Palmitate"[TIAB])

Nucleotides

Embase

#	Searches	Results
1	exp Meta Analysis/	144648
2	((meta adj analy$) or metaanalys$).tw.	168465
3	(systematic adj (review$1 or overview$1)).tw.	148021
4	or/1-3	291254
5	cancerlit.ab.	740
6	cochrane.ab.	79808
7	embase.ab.	82418
8	(psychlit or psyclit).ab.	996
9	(psychinfo or psycinfo).ab.	20842
10	(cinahl or cinhal).ab.	24293
11	science citation index.ab.	3312
12	bids.ab.	571
13	or/5-12	128593
14	reference lists.ab.	17025
15	bibliograph$.ab.	19151
16	hand-search$.ab.	7008
17	manual search$.ab.	4406
18	relevant journals.ab.	1313
19	or/14-18	43993
20	data extraction.ab.	19994
21	selection criteria.ab.	33126
22	20 or 21	50993
23	review.pt.	2353244
24	22 and 23	25406
25	letter.pt.	1013577
26	editorial.pt.	562504
27	animal/	1840342
28	human/	19481735
29	27 not (27 and 28)	79485
30	or/25-26,29	1655532
31	4 or 13 or 19 or 24	345681
32	31 not 30	336477
33	crossover-procedure/ or double-blind procedure/ or randomized controlled trial/ or single-blind procedure/ or (random* or factorial* or crossover* or cross over* or placebo* or (doubl* adj blind*) or (singl* adj blind*) or assign* or allocat* or volunteer*).tw.	1975974
34	32 or 33	2180399

35	exp nucleotide/ or "nucleic acids, nucleic acid components and their derivatives"/	538970
36	dietary nucleotide.m_titl.	21
37	nucleotid$.tw.	324666
38	infant nutrition.tw. or exp infant nutrition/ or nutritional support.tw. or exp nutritional support/ or infant$food.tw. or infant$diet.tw. or baby$food.tw. or baby$diet.tw. or exp baby food/ or exp artificial milk/	101863
39	exp cognition assessment/ or Cognition.tw. or exp cognition/ or exp social cognition/ or IQ.tw. or intelligence.tw. or exp Wechsler adult intelligence scale/ or exp intelligence quotient/ or exp Wechsler intelligence scale/ or exp "Wechsler preschool and primary scale of intelligence"/ or exp intelligence/ or exp Wechsler intelligence scale for children/ or exp intelligence test/ or exp emotional intelligence/ or Intelligence.mp. or exp Stanford-Binet Intelligence Scale/ or (neurodevelopment or cognitive).tw. or child$development.tw. or exp child development/ or exp neuropsychological test/ or exp child development/ or exp "Bayley Scales of Infant Development"/ or exp mental development/ or bayley.tw. or exp reading/ or exp school/ or exp learning disorder/ or exp educational status/ or exp education/ or exp achievement/ or exp academic achievement/ or school attainment.tw.	3302096
40	35 or 36 or 37	779918
41	34 and 38 and 39 and 40	17

MEDLINE®

MEDLINE terms for cognitive ability, study population, and infant nutrition (see above) and …

"Nucleotides"[Mesh] OR "Dietary nucleotides"[TIAB] OR "Nucleotides"[TIAB]

Supplement 2: Power calculations and primary outcomes for the original trials

Trial	Primary Outcomes	Delta / Power
NEP	Growth and neuro-development at 18 months postterm (Bayley Scales)	The target sample size per randomized group (113 infants) was calculated to permit detection of a 300-g difference in weight between groups at 9 months and a 400-g difference at 18 months (in each case the difference between the 10th and 25th centiles) at 5% significance and 70% to 80% power. This sample size would also permit detection of a 4-point (approximately 0.3 SD) difference in Bayley developmental indices.
NETSGA	Bayley Scales at 18 months	The primary hypothesis in respect to development was that there would be a one-third SD difference in Bayley MDI and PDI scores at 18 months between the 2 randomized groups. We calculated that, with 144 infants per group, we had 80% power at 5% significance to detect this difference, and a similar one-third SD difference in developmental scores at 9 months.
LCPUFAP	Bayley Scales at 18 months	The calculated sample size (100 infants per randomized group) permitted detection of a 0.4 standard deviation (SD) difference between diet groups with 80% power at 5% significance. Seventy- five subjects per randomized group (the approximate number seen at 18-month follow-up) would permit detection of a 0.46 SD difference between groups with 80% power at 5% significance.
LCPUFAT	Bayley Scales at 18 months	A five-point difference in the primary outcome, Bayley MDI at 18 months, was judged clinically relevant and was also chosen based on comparative studies of formula-fed infants versus breastfed infants, from which it has been hypothesised that the difference in developmental scores could relate to LCPUFAs in breast milk. With a targeted maximum of 142 individuals in each group, we were 80% likely to detect such a difference at the 5% level of significance (minimum recruitment was set at 111 individuals per group, to detect the hypothesised difference at 70% power). At 18 months, we had developmental data on 125 individuals in each group, in fact yielding over 95% power to detect a five-point difference, given that the actual SD was lower than that estimated.
IRONT	Bayley Scales at 18 months	The study was designed to have 80% power to detect an overall five-point (1/3 SD) difference in Bayley MDI and PDI at 5% significance between (1) the iron fortified follow-on formula and unfortified formula
PALMT	Stool hardness	Sample size was calculated to detect plausible differences in stool hardness and constipation between the study groups at 5% significance and 80% power. This sample size would enable a plausible 0.365-SD difference in radial BMC between randomly assigned groups to be detected at 5% significance and 80% power.
NUCLEOT	Diarrheal episodes	Sample size was initially calculated to detect a 0.5 SD difference in the number of diarrheal episodes between randomized formula-fed groups with 80% power at 5% significance. However, successful recruitment meant that the trial was continued beyond that originally planned to give a power of 0.4 SD difference in outcomes between the randomized groups at 80% power and $P < 0.05$.

Post-linkage delta estimation for primary outcome

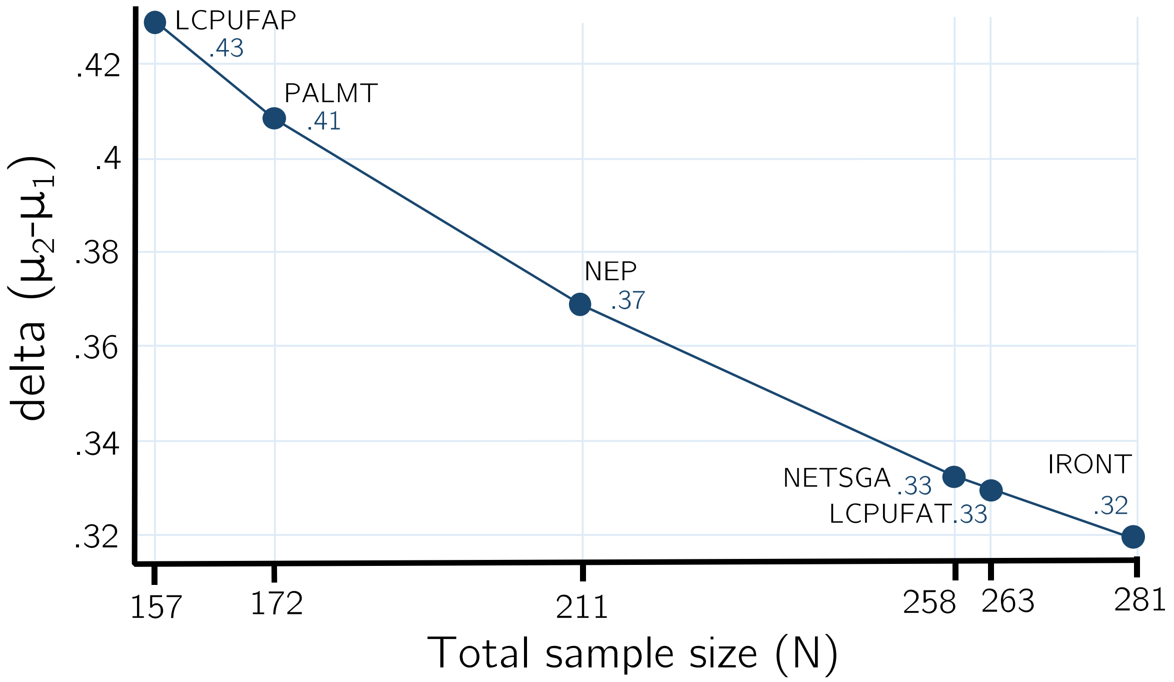

Parameters:
α = .05, μ_1 = 0, σ = 0.95, 1-β=0.8

The total (complete case) number of participants linked to the primary outcome in the NEP, NETSGA, LCPUFAP, LCPUFAT and IRONT trials was 211, 258, 157, 263, and 281 participants respectively. Assuming 80% power (1-β) and an average of 0.95 σ (achieved through covariate adjustment and multiple imputation in the primary analysis) these sample sizes could detect SD differences (δ) that were at least as large as 0.43 (LCPUFAP), 0.37 (NEP), 0.33 (NETSGA), 0.33 (LCPUFAT) and 0.32 SD (IRONT) respectively.

Supplement 3: Macronutrient compositions of intervention and control formulas in the different trials

	NEP		NETSGA		LCPUFAP		LCPUFAT		IRONT	
per 100 ml	Standard formula	Modified formula	Standard formula	Modified formula	Standard formula	Modified formula	Standard formula	Modified formula	Standard formula	Modified formula
Energy (kcal)	68	72	68	72	70	70	67	67	65	65
Protein (g)	1.45	1.85	1.45	1.85	2	2	1.5	1.5	2.5	2.5
Casein	0.56	0.72	0.56	0.72	0.8	0.8	0.6	0.6	1	1
Whey	0.89	1.13	0.89	1.13	1.2	1.2	0.9	0.9	1.5	1.5
Carbohydrate (g)	6.96	7.24	6.96	7.24	7.7	7.7	7.6	7.7	8	8
Fat (g)	3.82	3.96	3.82	3.96	3.5	3.5	3.4	3.4	2.8	2.8
Fatty acid composition (g/ 100 g fat)										
C10:0 capric					1.2	1.1	2.4	2		
C12:0 lauric					6.3	4.9	1.6	12.3		
C14:0 myristic					5.6	5.6	8.6	5.2		
C16:0 palmitic					25.8	26.3	23.3	25.6		
C18:0 stearic					8.2	8.5	10.2	4.6		
C18:1 oleic					32.6	32.9	32.7	29.7		
C18:2 n-6 linoleic					10.6	12	12.4	15.9		
C18:3 n-3 linolenic					0.7	0.6	1.1	1.4		
C20:4 n-6 AA					-	0.31	-	0.3		
C20:5 n-3 eicosapentaenoic					-	0.04	-	0.01		
C22:6 n-3 DHA					-	0.17	-	0.32		
Cholesterol					-	7.73	<0.5	0.8		

Information extracted from the original trial publications.

Supplement 4: Consistency of school outcomes with previous in-trial measurements. Pearson's correlation between cognitive measures (all trials combined)

	Bayley MDI	IQ age 6	English 11	Maths 11	English 16	Maths 16	IQ age 17	*Legend*
**Bayley MDI**	1							***1***
**IQ age 6**	0.3711	1						***0.8***
**English 11**	0.3342	0.5296	1					***0.4***
**Maths 11**	0.2671	0.4684	0.699	1				***0***
**English 16**	0.3012	0.5157	0.5945	0.5692	1			***-0.4***
**Maths 16**	0.2672	0.491	0.607	0.7731	0.7495	1		***-0.8***
**IQ age 17**	0.0728	0.4669	0.5664	0.7162	0.6231	0.7533	1	***-1***

Supplement 5: Robust standard errors used in this study

The trials showed heteroskedasticity and skewed residuals. While this does not bias regression coefficients, it makes the usual method to calculate standard errors (ordinary least squares, OLS) less efficient and can thereby affect the width of the confidence intervals. To address this issue we applied robust standard errors using Stata's *vce(robust)* option to all analyses for consistency:

$$var_r(\hat{\beta}_{OLS}) = (\frac{N}{N-K})(X'X)^{-1}\sum_{i=1}^{N}(X_iX_i'\hat{\epsilon}^2)(X'X)^{-1}$$

Equation 1: Sandwich estimator of variance used for calculation of robust standard errors

In the equation above, beta hat$_{OLS}$ is the sample estimator, and K are the cross-products of the variables X in the original regression equation. This equation describes that robust standard errors allow the variance of residuals to vary across the variables X. The standard errors are therefore consistent even if the residuals in the regression model do not have constant variance.

Supplement 6: Baseline characteristics at randomisation, by trial

	NEP		NETSGA		LCPUFAP		LCPUFAT		IRONT	
	Modified	Modified	Standard	Modified			Standard	Modified	Modified	Standard
Randomised, n	113	116	152	147	96	100	155	154	162	165
Average birth weight, min max (grams)	1378 (775-2160)	1359 (630-2020)	2532 (1400-3160)	2602 (1770-3160)	1329 (640-1850)	1352 (740-1800)	3648 (2950-4900)	3540 (2680-4930)	3493 (2495-5103)	3465 (2466-4706)
Average gestational age, min max (weeks)	30.7 (26-36)	30.8 (25-36)	39.0 (37-42)	39.4 (37-42)	30.3 (24-36)	30.3 (25-36)	40.1 (37-42)	40.0 (37-42)	39.8 (36-43)	39.9 (35-43)
Mother's age (years)	28.2 (16-41)	28.5 (17-44)	26.8 (15-42)	26.4 (14-42)	26.1 (16-39)	26.7 (17-39)	27.5 (17-44)	27.0 (18-41)	27.7 (17-40)	27.5 (15-39)
Infant sex										
Male, n (%)	53 (47%)	57 (50%)	74 (49%)	68 (46%)	42 (44%)	53 (53%)	82 (53%)	83 (54%)	82 (51%)	81 (49%)
Female, n (%)	60 (53%)	58 (50%)	78 (51%)	79 (54%)	54 (56%)	47 (47%)	73 (47%)	71 (46%)	79 (49%)	84 (51%)
Mother smoked during pregnancy										
No, n (%)	67 (61%)	74 (68%)	79 (55%)	67 (50%)	55 (57%)	60 (60%)	117 (77%)	110 (74%)	116 (73%)	111 (69%)
Yes, n (%)	42 (39%)	36 (32%)	64 (45%)	66 (50%)	41 (43%)	40 (40%)	35 (23%)	39 (26%)	44 (27%)	51 (31%)
Missing	4	6	9	14	0	0	3	5	2	3
Mother has degree										
No, n (%)	106 (94%)	97 (88%)	143 (94%)	139 (96%)	52 (91%)	47 (90%)	140 (92%)	145 (96%)	140 (88%)	147 (90%)
Yes, n (%)	7 (6%)	13 (12%)	9 (6%)	6 (4%)	5 (9%)	5 (10%)	13 (8%)	6 (4%)	20 (12%)	16 (10%)
Missing	0	6	0	2	39	48	2	3	2	2

	PALMT		NUCLEOT	
	Modified	Modified	Standard	Modified
Randomised, n	103	100	100	100
Average birth weight, min max (grams)	3575 (2640-4730)	3479 (2520-5400)	3455 (2210-4830)	3459 (2170-5360)
Average gestational age, min max (weeks)	40 (37-42)	39.9 (37-42)	39.4 (37-42)	39.2 (37-42)
Mother's age (years)	26.6 (15-40)	27.8 (17-42)	27 (16-44)	27 (16-40)
Infant sex				
Male, n (%)	66 (64%)	52 (52%)	56 (56%)	61 (61%)
Female, n (%)	37 (36%)	48 (48%)	44 (44%)	39 (39%)
Mother smoked during pregnancy				
No, n (%)	65 (63%)	74 (74%)	70 (71%)	58 (60%)
Yes, n (%)	38 (37%)	26 (26%)	28 (29%)	38 (40%)
Missing	0	0	1	1
Mother has degree				
No, n (%)	*	*	92 (92%)	92 (92%)
Yes, n (%)	*	*	8 (8%)	8 (8%)
Missing	76	70	0	0

** Suppressed due to small cell sizes.*

Supplement 7: Primary, Secondary and Sensitivity analyses

Primary analysis and sensitivity analyses of the primary outcome in the NEP trial showing mean and standard deviation of within-trial standardised GCSE Maths grade in the nutrient enriched and standard formula group and their mean differences with 95% confidence interval

NEP	Nutrient enriched formula group		Standard term formula group			
Primary outcome: within-trial standardised GCSE Maths grade	Mean	SD	Mean	SD	Mean difference	95% CI
Primary analysis						
MI adjusted (N_E=112, N_S=115)	0.01	0.95	-0.01	0.92	0.02	-0.22, 0.27
Sensitivity analyses						
MI unadjusted (N_E=112, N_S=115)	0.00	1.03	0.00	0.98	-0.01	-0.27, 0.25
Complete-case adjusted (N_E=98, N_S=100)	0.06	0.94	-0.03	0.89	0.09	-0.16, 0.35
Complete-case unadjusted (N_E=101, N_S=110)	-0.01	1.04	0.01	0.97	-0.02	-0.29, 0.26
MI adjusted national SD (N_E=112, N_S=115)	-0.42	0.99	-0.44	0.96	0.03	-0.23, 0.28

<u>Footnotes:</u> GCSE General Certificate of Secondary Education; MI multiple imputation of covariates and outcomes; NEP nutrient enriched preterm post-discharge trial; N_E Number of participants in the enriched formula group; N_S Number of participants in the standard formula group; SD standard deviation; adjusted for: infant sex, birth weight, gestational age, recruitment centre, maternal smoking during pregnancy, and maternal education at birth.

Primary analysis and sensitivity analyses of the primary outcome in the NETSGA trial showing mean and standard deviation of within-trial standardised GCSE Maths grade in the nutrient enriched and standard term formula group and their mean differences with 95% confidence interval

NETSGA	Nutrient enriched formula group		Standard term formula group			
Primary outcome: within-trial standardised GCSE Maths grade	Mean	SD	Mean	SD	Mean difference	95% CI
Primary analysis						
MI adjusted (N_E=152, N_S=147)	-0.05	0.97	0.05	0.99	-0.11	-0.33, 0.12
Sensitivity analyses						
MI unadjusted (N_E =152, N_S =147)	-0.02	1.00	0.02	1.00	0.04	-0.27, 0.19
Complete-case adjusted (N_E =122, N_S =114)	-0.10	1.00	0.09	1.05	-0.19	-0.46, 0.08
Complete-case unadjusted (N_E =130, N_S =128)	-0.03	1.01	0.03	0.99	-0.07	-0.31, 0.18
MI adjusted national (N_E =152, N_S =147)	-0.46	0.93	-0.36	095	-0.10	-0.32, 0.12

<u>Footnotes:</u> GCSE General Certificate of Secondary Education; MI multiple imputation of covariates and outcomes; NETSGA nutrient enriched term small-for-gestational-age trial; N_E Number of participants in the enriched formula group; N_S Number of participants in the standard formula group; SD standard deviation; adjusted for: infant sex, birth weight, gestational age, recruitment centre, maternal smoking during pregnancy, and maternal education at birth.

Primary analysis and sensitivity analyses of the primary outcome in the LCPUFAP trial showing mean and standard deviation of within-trial standardised GCSE Maths grade in the LCPUFA preterm and standard preterm formula group and their mean differences with 95% confidence interval

LCPUFAP	LCPUFA formula (0.17% DHA and 0.31% AA / total fat)		Standard preterm formula group (no DHA or AA)			
Primary outcome: within-trial standardised GCSE Maths grade	Mean	SD	Mean	SD	Mean difference	95% CI
Primary analysis						
MI adjusted (N_L=92, N_S=100)	-0.10	0.89	0.09	0.97	-0.19	-0.46, 0.08
Sensitivity analyses						
MI unadjusted (N_L=92, N_S=100)	-0.10	0.97	0.10	1.03	-0.20	-0.48, 0.09
Complete-case adjusted (N_L=48, N_S=44)	-0.02	0.92	0.20	1.00	-0.22	-0.62, 0.19
Complete-case unadjusted (N_L=74, N_S=83)	-0.09	0.97	-0.08	1.03	-0.17	-0.49, 0.14
MI adjusted national SD (N_L=92, N_S=100)	-0.75	0.93	-0.55	1.02	-0.20	-0.48, 0.09

Footnotes: GCSE General Certificate of Secondary Education; MI multiple imputation of covariates and outcomes; LCPUFAP long-chain polyunsaturated fatty acid supplemented formula for preterm babies trial; N_L Number of participants in the LCPUFA formula group; N_S Number of participants in the standard formula group; SD standard deviation; adjusted for: infant sex, birth weight, gestational age, recruitment centre, maternal smoking during pregnancy, and maternal education at birth.

Primary analysis and sensitivity analyses of the primary outcome in the LCPUFAT trial showing mean and standard deviation of within-trial standardised GCSE Maths grade in the LCPUFA and standard formula group and their mean differences with 95% confidence interval

LCPUFAT	LCPUFA formula (0.32% DHA and 0.30% AA /total fat)		Standard term formula (no DHA or AA)			
Primary outcome: within-trial standardised GCSE Maths grade	Mean	SD	Mean	SD	Mean difference	95% CI
Primary analysis						
MI adjusted (N_L=155, N_S=154)	-0.07	0.97	0.07	0.97	-0.14	-0.36, 0.08
Sensitivity analyses						
MI unadjusted (N_L=155, N_S=154)	-0.04	1.02	0.04	0.99	-0.07	-0.30, 0.15
Complete-case adjusted (N_L=122 N_S=130)	-0.02	0.96	0.07	0.98	-0.09	-0.33, 0.16
Complete-case unadjusted (N_L=127, N_S=136)	-0.02	1.01	0.02	0.99	-0.04	-0.28, 0.21
MI adjusted national SD (N_L=155, N_S=154)	-0.36	0.85	-0.23	0.84	-0.12	-0.31, 0.07

Footnotes: GCSE General Certificate of Secondary Education; MI multiple imputation of covariates and outcomes; LCPUFAT long-chain polyunsaturated fatty acid supplemented formula for term babies trial; N_L Number of participants in the LCPUFA formula group; N_S Number of participants in the standard formula group; SD standard deviation; adjusted for: infant sex, birth weight, gestational age, recruitment centre, maternal smoking during pregnancy, and maternal education at birth.

Primary analysis and sensitivity analyses of the primary outcome in the IRONT trial showing mean and standard deviation of within-trial standardised GCSE Maths grade in the high-iron and low-iron formula group and their mean differences with 95% confidence interval

IRONT	High iron (12mg/dl) formula		Low iron (0.9mg/dl) formula			
Primary outcome: within-trial standardised GCSE Maths grade	Mean	SD	Mean	SD	Mean difference	95% CI
Primary analysis						
MI adjusted (N_{HI}=162, N_{LI}=165)	-0.06	0.95	0.06	0.83	-0.12	-0.31, 0.07
Sensitivity analyses						
MI unadjusted (N_{HI}=162, N_{LI}=165)	-0.05	1.07	0.05	0.93	-0.10	-0.44, 0.00
Complete-case adjusted (N_{HI}=137, N_{LI}=140)	-0.05	0.98	0.04	0.86	-0.09	-0.31, 0.13
Complete-case unadjusted (N_{HI}=138, N_{LI}=143)	-0.02	1.06	0.02	0.94	-0.05	-0.28, 0.19
MI adjusted national SD (N_{HI}=162, N_{LI}=165)	-0.19	0.89	-0.07	0.78	-0.12	-0.30, 0.07

Footnotes: GCSE General Certificate of Secondary Education; MI multiple imputation of covariates and outcomes; IRONT iron trial; N_{HI} Number of participants in the high iron formula group; N_{LI} Number of participants in the low-iron formula group; SD standard deviation; adjusted for: infant sex, birth weight, gestational age, recruitment centre, maternal smoking during pregnancy, and maternal education at birth.

Primary analysis and sensitivity analyses of the primary outcome in the PALMT trial showing mean and standard deviation of within-trial standardised GCSE Maths grade in the 50% sn-2 palmitate and 12% sn-2 palmitate formula group and their mean differences with 95% confidence interval

**PALMT**	Sn-2 palmitate formula		Standard palmitate formula			
Primary outcome: within-trial standardised GCSE Maths grade	*Mean SD-score*	*SD*	*Mean SD-score*	*SD*	*Standardised mean difference*	*95% CI*
Primary analysis						
MI adjusted (N_P=103, N_S=100)	-0.04	1.03	0.05	0.96	-0.09	-0.37, 0.19
Sensitivity analyses						
MI unadjusted (N_P=103, N_S=100)	-0.07	1.04	0.07	0.96	-0.14	-0.41, 0.14
Complete-case adjusted (N_P=27, N_S=27)	0.21	1.05	0.36	0.81	-0.16	-0.71, 0.39
Complete-case unadjusted (N_P=91, N_S=81)	-0.05	1.03	0.05	0.96	-0.10	-0.40, 0.20
MI adjusted national SD (N_P=103, N_S=100)	-0.27	1.06	0.18	0.98	-0.09	-0.37, 0.19

Footnotes: GCSE *General Certificate of Secondary Education*; MI *multiple imputation of covariates and outcomes*; PALM *Sn-2 Palmitate trial*; N_P *Number of participants in the sn-2 palmitate formula group*; N_S *Number of participants in the standard palmitate formula group; SD standard deviation;* adjusted for: infant sex, birth weight, gestational age, recruitment centre, maternal smoking during pregnancy, and maternal education at birth; National reference distribution: UK GCSE Maths grades and SD pooled for 2008/09 to 2011/12.

The English national average and standard deviation during the respective year was derived with data from: https://www.gov.uk/government/collections/statistics-gcses-key-stage-4

Secondary outcomes in the NEP trial showing mean differences in modified vs standard formula of GCSE English language exam as within-trial SD-scores, of Maths and English reading exams as within-trial SD-scores at age 11 years (KS2, final year of primary school) and odds ratios in modified vs standard formula of receiving five or more GCSE grades A* to C (including Maths and English) and of ever being eligible for special educational needs (SEN) support

NEP<br>N_I=112, N_C=115	Nutrient enriched formula		Standard formula			
Within-trial standardised grades:	Mean	SD	Mean	SD	Mean difference	95% CI
GCSE English (age 16)	0.00	0.87	0.00	0.90	0.00	-0.23, 0.23
KS2 Maths (age 11)	0.01	0.96	-0.01	0.92	0.02	-0.22, 0.27
KS2 English (age 11)	-0.05	0.91	0.05	0.93	-0.09	-0.33, 0.15
Other secondary outcomes:					Odds ratio	95% CI
Ever qualified for special educational needs					1.29	0.72, 2.32
5+ GCSE grades ≥C					1.27	0.70, 2.29

<u>Footnotes:</u> GCSE General Certificate of Secondary Education; NEP nutrient enriched preterm post-discharge trial; N_E Number of participants in the enriched formula group; N_S Number of participants in the standard formula group; SD standard deviation; adjusted for: infant sex, birth weight, gestational age, recruitment centre, maternal smoking during pregnancy, and maternal education at birth; covariates and outcomes imputed for missing participants who have not died.

Secondary outcomes in the NETSGA trial showing mean differences in modified vs standard formula of GCSE English language exam as within-trial SD-scores, of Maths and English reading exams as within-trial SD-scores at age 11 years (KS2, final year of primary school) and odds ratios in modified vs standard formula of receiving five or more GCSE grades A* to C (including Maths and English) and of ever being eligible for special educational needs (SEN) support

NETSGA<br>N_I=152, N_C=147	Nutrient enriched		Standard formula			
Within-trial standardised grades:	Mean	SD	Mean	SD	Mean difference	95% CI
GCSE English (age 16)	-0.06	0.97	0.06	0.94	-0.12	-0.34, 0.10
KS2 Maths (age 11)	-0.09	1.01	0.09	0.98	-0.18	-0.41, 0.05
KS2 English (age 11)	-0.10	0.98	0.10	1.01	-0.20	-0.43, 0.03
Other secondary outcomes:					Odds ratio	95% CI
Ever qualified for special educational needs					1.49	0.90, 2.47
5+ GCSE grades ≥C					1.00	0.60, 1.71

<u>Footnotes:</u> GCSE General Certificate of Secondary Education; NETSGA nutrient enriched term small-for-gestational-age trial; N_E Number of participants in the enriched formula group; N_S Number of participants in the standard formula group; SD standard deviation; adjusted for: infant sex, birth weight, gestational age, recruitment centre, maternal smoking during pregnancy, and maternal education at birth; covariates and outcomes imputed for missing participants who have not died.

Secondary outcomes in the LCPUFAP trial showing mean differences in modified vs standard formula of GCSE English language exam as within-trial SD-scores, of Maths and English reading exams as within-trial SD-scores at age 11 years (KS2, final year of primary school) and odds ratios in modified vs standard formula of receiving five or more GCSE grades A* to C (including Maths and English) and of ever being eligible for special educational needs (SEN) support

LCPUFAP<br>N_I=92, N_C=100	LCPUFA formula (0.17% DHA and 0.31% AA/ total fat)		Standard preterm formula (no DHA or AA)			
Within-trial standardised grades:	Mean	SD	Mean	SD	Mean difference	95% CI
GCSE English (age 16)	-0.11	0.88	0.10	0.93	-0.21	-0.48, 0.06
KS2 Maths (age 11)	-0.19	0.92	0.18	0.96	-0.37	-0.64, -0.09
KS2 English (age 11)	-0.15	0.96	0.14	0.91	-0.29	-0.56, -0.01
Other secondary outcomes:					Odds ratio	95% CI
Ever qualified for special educational needs					1.34	0.68, 2.64
5+ GCSE grades ≥C					0.65	0.32, 1.31

<u>Footnotes:</u> GCSE General Certificate of Secondary Education; LCPUFAP long-chain polyunsaturated fatty acid supplemented formula for preterm babies trial; N_L Number of participants in the LCPUFA formula group; N_S Number of participants in the standard formula group; SD standard deviation; adjusted for: infant sex, birth weight, gestational age, recruitment centre, maternal smoking during pregnancy, and maternal education at birth; covariates and outcomes imputed for missing participants who have not died.

Secondary outcomes in the LCPUFAT trial showing mean differences in modified vs standard formula of GCSE English language exam as within-trial SD-scores, of Maths and English reading exams as within-trial SD-scores at age 11 years (KS2, final year of primary school) and odds ratios in modified vs standard formula of receiving five or more GCSE grades A* to C (including Maths and English) and of ever being eligible for special educational needs (SEN) support

LCPUFAT<br>N_I=155, N_C=154	LCPUFA formula (0.17% DHA and 0.31% AA/ total fat)		Standard preterm formula (no DHA or AA)			
Within-trial standardised grades:	Mean	SD	Mean	SD	Mean difference	95% CI
GCSE English (age 16)	-0.06	0.97	0.06	0.94	-0.13	-0.35, 0.09
KS2 Maths (age 11)	-0.15	0.99	0.15	0.93	-0.29	-0.51, -0.08
KS2 English (age 11)	-0.16	0.93	0.16	0.93	-0.33	-0.53, -0.11
Other secondary outcomes:					Odds ratio	95% CI
Ever qualified for special educational needs					1.29	0.78,2.14
5+ GCSE grades ≥C					0.69	0.41, 1.16

<u>Footnotes:</u> GCSE General Certificate of Secondary Education; LCPUFAT long-chain polyunsaturated fatty acid supplemented formula for term babies trial; N_L Number of participants in the LCPUFA formula group; N_S Number of participants in the standard formula group; SD standard deviation; adjusted for: infant sex, birth weight, gestational age, recruitment centre, maternal smoking during pregnancy, and maternal education at birth; covariates and outcomes imputed for missing participants who have not died.

Secondary outcomes in the NETSGA trial showing mean differences in modified vs standard formula of GCSE English language exam as IRONT trial SD-scores, of Maths and English reading exams as within-trial SD-scores at age 11 years (KS2, final year of primary school) and odds ratios in modified vs standard formula of receiving five or more GCSE grades A* to C (including Maths and English) and of ever being eligible for special educational needs (SEN) support

IRONT<br>N_I=162, N_C=165	High iron (12mg/dl) formula		Low iron (0.9mg/dl) formula			
Within-trial standardised grades:	Mean	SD	Mean	SD	Mean difference	95% CI
GCSE English (age 16)	-0.08	0.81	0.07	0.85	-0.15	-0.33, 0.03
KS2 Maths (age 11)	-0.04	0.94	0.04	0.92	-0.08	-0.29, 0.12
KS2 English (age 11)	0.01	0.93	-0.01	0.90	0.02	-0.18, 0.22
Other secondary outcomes:					Odds ratio	95% CI
Ever qualified for special educational needs					1.32	0.80, 2.18
5+ GCSE grades ≥C					1.30	0.67, 2.52

<u>Footnotes:</u> GCSE General Certificate of Secondary Education; IRONT iron trial; N_{HI} Number of participants in the high iron formula group; N_{LI} Number of participants in the low-iron formula group; SD standard deviation; adjusted for: infant sex, birth weight, gestational age, recruitment centre, maternal smoking during pregnancy, and maternal education at birth; covariates and outcomes imputed for missing participants who have not died.

Secondary outcomes in the NETSGA trial showing mean differences in modified vs standard formula of GCSE English language exam as IRONT trial SD-scores, of Maths and English reading exams as within-trial SD-scores at age 11 years (KS2, final year of primary school) and odds ratios in modified vs standard formula of receiving five or more GCSE grades A* to C (including Maths and English) and of ever being eligible for special educational needs (SEN) support

IRONT<br>N_I=162, N_C=165	High iron (12mg/dl) formula		Low iron (0.9mg/dl) formula			
Within-trial standardised grades:	Mean	SD	Mean	SD	Mean difference	95% CI
GCSE English (age 16)	-0.08	0.81	0.07	0.85	-0.15	-0.33, 0.03
KS2 Maths (age 11)	-0.04	0.94	0.04	0.92	-0.08	-0.29, 0.12
KS2 English (age 11)	0.01	0.93	-0.01	0.90	0.02	-0.18, 0.22
Other secondary outcomes:					Odds ratio	95% CI
Ever qualified for special educational needs					1.32	0.80, 2.18
5+ GCSE grades ≥C					1.30	0.67, 2.52

<u>Footnotes:</u> GCSE General Certificate of Secondary Education; IRONT iron trial; N_{HI} Number of participants in the high iron formula group; N_{LI} Number of participants in the low-iron formula group; SD standard deviation; adjusted for: infant sex,

birth weight, gestational age, recruitment centre, maternal smoking during pregnancy, and maternal education at birth; covariates and outcomes imputed for missing participants who have not died.

Secondary outcomes in the NETSGA trial showing mean differences in modified vs standard formula of GCSE English language exam as IRONT trial SD-scores, of Maths and English reading exams as within-trial SD-scores at age 11 years (KS2, final year of primary school) and odds ratios in modified vs standard formula of receiving five or more GCSE grades A* to C (including Maths and English) and of ever being eligible for special educational needs (SEN) support

IRONT N_I=162, N_C=165	High iron (12mg/dl) formula		Low iron (0.9mg/dl) formula			
Within-trial standardised grades:	Mean	SD	Mean	SD	Mean difference	95% CI
GCSE English (age 16)	-0.08	0.81	0.07	0.85	-0.15	-0.33, 0.03
KS2 Maths (age 11)	-0.04	0.94	0.04	0.92	-0.08	-0.29, 0.12
KS2 English (age 11)	0.01	0.93	-0.01	0.90	0.02	-0.18, 0.22
Other secondary outcomes:					Odds ratio	95% CI
Ever qualified for special educational needs					1.32	0.80, 2.18
5+ GCSE grades ≥C					1.30	0.67, 2.52

<u>Footnotes:</u> GCSE General Certificate of Secondary Education; IRONT iron trial; N_{HI} Number of participants in the high iron formula group; N_{LI} Number of participants in the low-iron formula group; SD standard deviation; adjusted for: infant sex, birth weight, gestational age, recruitment centre, maternal smoking during pregnancy, and maternal education at birth; covariates and outcomes imputed for missing participants who have not died.

Secondary outcomes in the PALMT trial showing mean differences in modified vs standard formula of GCSE English language exam as PALMT trial SD-scores, of Maths and English reading exams as within-trial SD-scores at age 11 years (KS2, final year of primary school) and odds ratios in modified vs standard formula of receiving five or more GCSE grades A* to C (including Maths and English) and of ever being eligible for special educational needs (SEN) support

**PALMT** N_I=103, N_C=100	Sn-2 palmitate formula		Standard palmitate formula			
Within-trial standardised grades:	*Mean*	*SD*	*Mean*	*SD*	*SMD*	*95% CI*
GCSE English (age 16)	-0.03	0.95	0.03	0.93	-0.06	-0.33, 0.21
KS2 Maths (age 11)	-0.02	1.00	0.02	0.99	-0.03	-0.32, 0.25
KS2 English (age 11)	0.08	0.95	-0.08	1.03	0.15	-0.13, 0.43
Other secondary outcomes:					*Odds ratio*	*95% CI*
Ever qualified for special educational needs					0.81	0.42, 1.53
5+ GCSE grades ≥C					1.30	0.67, 2.52

<u>Footnotes</u>: GCSE *General Certificate of Secondary Education;* PALM *Sn-2 Palmitate trial*; N_P *Number of participants in the sn-2 palmitate formula group*; N_S *Number of participants in the standard palmitate formula group;* SD *standard deviation;* SMD *standardised mean difference;* adjusted for: infant sex, birth weight, gestational age, recruitment centre, maternal smoking during pregnancy, and maternal education at birth; covariates and outcomes imputed for missing participants who have not died.

Secondary outcomes in the NUCLEOT trial showing mean differences in modified vs standard formula of Maths and English reading exams as within-trial SD-scores at age 11 years (KS2, final year of primary school) and odds ratios in modified vs standard formula of ever being eligible for special educational needs (SEN) support

NUCLEO N_I=99, N_C=97		
Within-trial standardised grades:	SMD	95% CI
KS2 Maths (age 11)	0.17	-0.12, 0.46
KS2 English (age 11)	0.00	-0.28, 0.28
Other secondary outcomes:	Odds ratio	95% CI
Ever qualified for special educational needs	0.50	0.25, 1.01

References

1. Young L, Embleton ND, McGuire W. Nutrient-enriched formula versus standard formula for preterm infants following hospital discharge. Cochrane Database of Systematic Reviews. 2016;12(12):CD004696.
2. Singhal A, Kennedy K, Lanigan J, Fewtrell M, Cole TJ, Stephenson T, et al. Nutrition in infancy and long-term risk of obesity: evidence from 2 randomized controlled trials. The American journal of clinical nutrition. 2010;92(5):1133-44.
3. Embleton ND, Wood CL, Pearce MS, Brunskill G, Grahame V. Early diet in preterm infants and later cognition: 10-year follow-up of a randomized controlled trial. Pediatric Research. 2021.
4. Sun H, Como PG, Downey LC, Murphy D, Ariagno RL, Rodriguez W. Infant formula and neurocognitive outcomes: impact of study end-point selection. J Perinatol. 2015;35(10):867-74.
5. Gibson RA, Makrides M. Long-chain polyunsaturated fatty acids in breast milk: are they essential? Advances in experimental medicine and biology. 2001;501:375-83.
6. Neuringer M, Connor WE. n-3 fatty acids in the brain and retina: evidence for their essentiality. Nutrition Reviews. 1986;44(9):285-94.
7. Woods J, Ward G, Salem N. Is Docosahexaenoic Acid Necessary in Infant Formula? Evaluation of High Linolenate Diets in the Neonatal Rat. Pediatric Research. 1996;40(5):687-94.
8. Lapillonne A, Groh-Wargo S, Lozano Gonzalez CH, Uauy R. Lipid Needs of Preterm Infants: Updated Recommendations. The Journal of pediatrics. 2013;162(3, Supplement):S37-S47.
9. Jasani B, Simmer K, Patole SK, Rao SC. Long chain polyunsaturated fatty acid supplementation in infants born at term. The Cochrane database of systematic reviews. 2017;3:CD000376.
10. Moon K, Rao S, Patole S, Simmer K, Schulzke S. Longchain polyunsaturted fatty acid supplementation in preterm infants: Updated cochrane review. Journal of Paediatrics and Child Health. 2017;53 (Supplement 2):69-70.
11. Verfuerden ML, Dib S, Jerrim J, Fewtrell M, Gilbert RE. Effect of long-chain polyunsaturated fatty acids in infant formula on long-term cognitive function in childhood: A systematic review and meta-analysis of randomised controlled trials. PLOS ONE. 2020;15(11):e0241800.
12. Halterman JS, Kaczorowski JM, Aligne CA, Auinger P, Szilagyi PG. Iron Deficiency and Cognitive Achievement Among School-Aged Children and Adolescents in the United States. Pediatrics. 2001;107(6):1381-6.
13. Lozoff B, Georgieff MK. Iron Deficiency and Brain Development. Seminars in Pediatric Neurology. 2006;13(3):158-65.
14. Jaeggi T, Kortman GAM, Moretti D, Chassard C, Holding P, Dostal A, et al. Iron fortification adversely affects the gut microbiome, increases pathogen abundance and induces intestinal inflammation in Kenyan infants. Gut. 2015;64(5):731-42.
15. Gahagan S, Delker E, Blanco E, Burrows R, Lozoff B. Randomized Controlled Trial of Iron-Fortified versus Low-Iron Infant Formula: Developmental Outcomes at 16 Years. The Journal of pediatrics. 2019;212:124-30.e1.
